# Heterogeneous Redistribution of Facial Subcategory Information Within and Outside the Face-Selective Domain in Primate Inferior Temporal Cortex

**DOI:** 10.1093/cercor/bhx342

**Published:** 2018-01-10

**Authors:** Naohisa Miyakawa, Kei Majima, Hirohito Sawahata, Keisuke Kawasaki, Takeshi Matsuo, Naoki Kotake, Takafumi Suzuki, Yukiyasu Kamitani, Isao Hasegawa

**Affiliations:** 1 Department of Physiology, Niigata University School of Medicine, Niigata, Japan; 2 Center for Transdisciplinary Research, Niigata University, Niigata, Japan; 3 Department of Functional Brain Imaging Research, National Institutes of Quantum and Radiological Science and Technology, Chiba, Japan; 4 Department of Intelligence Science and Technology, Graduate School of Informatics, Kyoto University, Kyoto, Japan; 5 Department of Neurosurgery, School of Medicine, University of Tokyo, Bunkyo-ku, Japan; 6 Department of Fisheries Distribution and Management, National Fisheries University, Shimonoseki, Japan; 7 Center for Information and Neural Networks, National Institute of Information and Communications Technology, Suita, Japan; 8 Department of Neuroinformatics, ATR Computational Neuroscience Laboratories, Keihanna Science City, Japan

**Keywords:** decoding, electrocorticogram, face, local field potential, multi-electrode array

## Abstract

The inferior temporal cortex (ITC) contains neurons selective to multiple levels of visual categories. However, the mechanisms by which these neurons collectively construct hierarchical category percepts remain unclear. By comparing decoding accuracy with simultaneously acquired electrocorticogram (ECoG), local field potentials (LFPs), and multi-unit activity in the macaque ITC, we show that low-frequency LFPs/ECoG in the early evoked visual response phase contain sufficient coarse category (e.g., face) information, which is homogeneous and enhanced by spatial summation of up to several millimeters. Late-induced high-frequency LFPs additionally carry spike-coupled finer category (e.g., species, view, and identity of the face) information, which is heterogeneous and reduced by spatial summation. Face-encoding neural activity forms a cluster in similar cortical locations regardless of whether it is defined by early evoked low-frequency signals or late-induced high-gamma signals. By contrast, facial subcategory-encoding activity is distributed, not confined to the face cluster, and dynamically increases its heterogeneity from the early evoked to late-induced phases. These findings support a view that, in contrast to the homogeneous and static coarse category-encoding neural cluster, finer category-encoding clusters are heterogeneously distributed even outside their parent category cluster and dynamically increase heterogeneity along with the local cortical processing in the ITC.

## Introduction

Humans recognize individual objects by sorting them into multiple categories, which are often hierarchically structured. For example, a dog is recognized more specifically by its breed (e.g., Dalmatian) or more vaguely as a 4-legged animal, depending on the context. The present study aims to clarify the mechanisms by which the hierarchical structure of perceptual categories is reflected in the co-ordinated activity of neuronal populations in the brain. Accumulating evidence suggests that the inferior temporal cortex (ITC) in the ventral visual system of primates contains neural correlates of different levels of category recognition, ranging from ordinate-level categorization (Rosch 1978; Wang et al. 1996; Haxby et al. 2001; Hung et al. 2005; Kiani et al. 2007; Sato et al. 2013) to subordinate-level discrimination (Wang et al. 1996; Sugase et al. 1999; Kreiman et al. 2000; Quiroga et al. 2005; Kriegeskorte et al. 2008; Huth et al. 2012; Sato et al. 2013). Neuroimaging and electrophysiological studies have indicated that there is a mosaic of brain regions highly selective to distinct coarse categories, such as faces (Kanwisher et al. 1997; Tsao et al. 2003; Tsao et al. 2006; ; Sato et al. 2013), places (Epstein and Kanwisher 1998), and other objects (Bell et al. 2011; Ku et al. 2011; Sato et al. 2013) in the ITC. Animal studies have also shown that neuronal activity in the ITC is selective to different subcategories of face, such as faces from specific viewing angles (Wang et al. 1996) and faces of particular animal species (Sato et al. 2013). Some neurons in the anterior/medial temporal lobe have been found to be sensitive to facial identities regardless of the viewing angle (Quiroga et al. 2005). However, there has been little evidence about the ways in which neuronal representations of facial subcategories (facial species, views, and identity) are spatially and temporally organized, or how subcategory-encoding neuronal clusters, if any, are topologically related to the coarser face category-selective cluster in the ITC. The present study examined these questions in 3 steps.

First, we investigated whether neurons selective to facial subcategories form discrete clusters in the ITC. Specifically, to estimate the spatiotemporal clustering of neuronal activity representing ordinate (face) and subordinate (facial view, species, and identity) categories, we test whether multichannel patterns of multi-unit activity (MUA), local field potentials (LFPs), or electrocorticogram (ECoG) across a region in the anterior ITC contain information sufficient to predict distinct levels of the sought category, using a decoding-based approach. The scale of spatiotemporal summation has been shown to vary across spiking activities, LFPs, and ECoG by direct comparisons in rodent (Helmchen et al. 1999), cat (Contreras and Steriade 1995), and macaque cortices (Belitski et al. 2008; Buzsaki et al. 2012). Thus, the difference in the amount of category information extractable from respective recorded data would be expected to reflect the spatiotemporal scale and uniformity of category-specific neuronal clusters (Kamitani and Tong 2005). Furthermore, comparison of decoding accuracy with simultaneously acquired MUA, LFPs, and ECoG may enable a reasonable prediction about whether category information of a particular level is enhanced or reduced by spatial summation up to several millimeters and could aid the understanding of spatiotemporal clustering of the neuronal activity encoding different levels of category information in the ITC. For simultaneous acquisition of MUA, LFPs, and ECoG data, we combined a high-density surface field potential recording technique recently established in our laboratory (Matsuo et al. 2011; Toda et al. 2011; Nakahara et al. 2016) and a high-density microelectrode-array technique (Dotson et al. 2015).

Second, we estimated the frequency dependency and temporal stability of category-specific IT architecture, again using the decoding approach. Previous studies have indicated that individual IT neurons can change their category preferences over the visual response time course, developing a preference for finer categories (Sugase et al. 1999) and sharpening stimulus tuning (Tamura and Tanaka 2001; Brincat and Connor 2006). However, little is known about whether category-selective IT architecture defined by frequency-specific synchronous activity is stable or changes dynamically during the visual response. In early visual cortices, LFPs, particularly the stimulus-locked early theta and initial transient high-gamma power (“evoked activity”), mainly reflect the initial synaptic inputs to the granular cortical layer and the immediately following polysynaptic activity within the local recorded region (Mitzdorf 1985, 1987; Belitski et al. 2008). In contrast, high-gamma power in the later period (“induced activity”) reflects further processing in the local recurrent network (Buzsaki et al. 2012). Recent studies have reported that low-frequency LFPs carry spike firing-independent information in the primate primary visual cortex (V1; Belitski et al. 2008). In the current study, we examined whether high-frequency LFPs carry category-selective information that is tightly coupled with output spike selectivity, and whether low-frequency LFPs carry spike-independent category information in the ITC, as in V1. For this purpose, we compared category-level–specific information embedded in early evoked LFPs, late-induced LFPs, and MUA. Further, by examining the time–frequency specificity of the decoded signals, we tested whether elaboration of categorical cortical representations through local processing within the ITC, from the early “evoked” low-frequency–dominant architecture to the late “induced” high-frequency–dominant architecture, depends on the level of category.

Interpretation of the spatial scale of different category clusters in the ITC by differences in decoding accuracy with LFPs, MUAs, and ECoGs is reasonable (see comparison of LFP, but not ECoG, to multiple levels of spatially summated MUA signal in macaque IT) (Kreiman et al. 2006), but suggestive. Thus, in the third part of the paper, we aimed to clarify the spatial and temporal factors contributing to the category-level–dependent “spatiotemporal neuronal clusters” identified by the decoding analyses. Specifically, we focused on the LFP-based IT architecture encoding the face category and its subcategories. We created coarse (ordinate) category including faces, and facial species, view, and identity (subordinate) category selectivity maps from early evoked low-frequency LFPs and late-induced high-frequency LFPs, and examined whether the actual clustering of neuronal activity with similar category selectivity in cortical space contributed to “spatiotemporal clusters.” Further, to clarify the spatial relationship between cortical representations of a parent category and its subcategories, we tested whether the clustering of facial subcategory-selective channels and the strength of channel-wise subcategory selectivity are greater within the parent face category domain than outside. It has been previously reported that the spatial reach of the recorded neural signal depends not only on spatial configuration but also on the temporal coherence of the source signals because phase matching of synaptic activity affects the spatial summation of the signal (Linden et al. 2011; Einevoll et al. 2013). By analyzing the phase of evoked LFPs, we investigated whether spatial patterns and temporal coherence both contribute to the separation of species and view category information.

## Materials and Methods

### Animals

Two Japanese macaque monkeys (*Macaca fuscata*), 1 male (9.5 kg) and 1 female (5.7 kg), provided by the National BioResource Project “Japanese Monkeys” by MEXT Japan, were used for the experiments. All experiments were performed in accordance with the National Institutes of Health Guidelines for the Care and Use of Laboratory Animals. The experimental protocol was approved by the Niigata University Institutional Animal Care and Use Committee.

### Task and Stimuli

**Figure 1.**
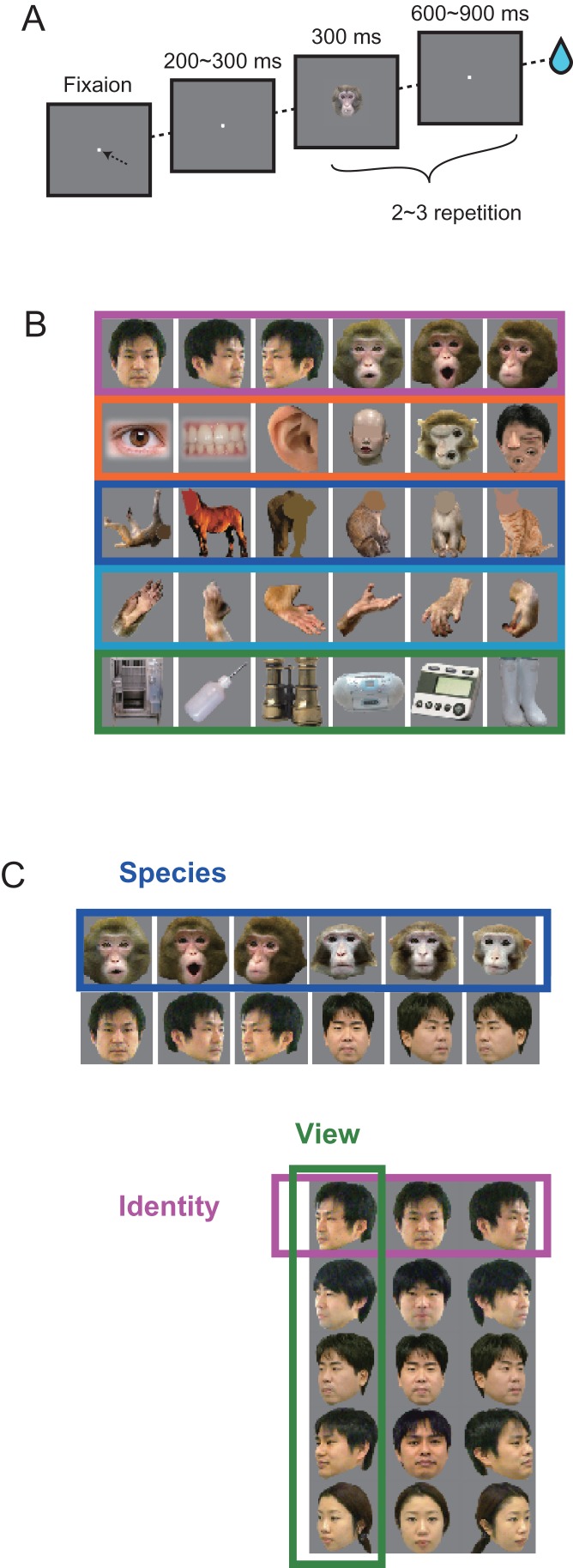
Visual stimuli and the presentation paradigm. (*A*) Two to three stimuli were presented during a passive fixation task. (B) Stimulus set consisting of different categorical levels. The coarse category set included faces, face parts, bodies, body parts, and inanimate objects. The fine categories were the face images used in the coarse category subdivided into species, views, and identities. The species category set included human faces and monkey faces with frontal view angle and gaze direction. The view and identity sets included human faces of 5 identities in 3 viewing angles. (*C*) Stimulus set used for monkey C, with the coarse category structure for faces, face parts, bodies, body parts, and inanimate objects that correspond to the coarse categories of the stimulus set for monkey H shown in (*B*).

Monkeys were trained in a visual fixation task (Fig. 1*A*) to keep their gaze within a 2–3° fixation window, while a 0.2–0.3° fixation spot was displayed on a 22-inch cathode ray tube monitor (Mitsubishi Electric, Tokyo, Japan) at a viewing distance of 57 cm. After 300 ms of stable fixation, a stimulus image was presented for 300 ms, followed by a 600–900-ms blank interval. Two or three stimuli were successively presented in a single fixation session. Monkeys passively viewed the stimulus set and were rewarded with a drop of apple juice for maintaining fixation over the entire duration of the trial. Eye movements were captured with an infrared camera system (i-rec https://staff.aist.go.jp/k.matsuda/iRecHS2/index_e.html, date last accessed December 14, 2017) at a sampling rate of 60 Hz. The behavior of animals was controlled by an in-house program written in MATLAB (Mathworks, Natick, USA) and OpenEx (TuckerDavisTechnologies (TDT), Alachua, USA) running on a Windows PC and a multicore digital signal processor (RZ2, TDT), which make up a multichannel acquisition system (System3, TDT). Stimuli were presented via the ViSaGe System (CambridgeResearchSystem, Rochester, UK), which was controlled by another in-house MATLAB program that also feeds stimulus timing to TDT with a transistor-transistor-logic (TTL) pulse.

### Anatomical MRI

To acquire structural images of the monkey brains, we used a 4.7-T MRI scanner with 100-mT/m actively shielded gradient coils and a volume radiofrequency (RF) coil (Biospec 47/40; Bruker, Ettlingen, Germany). High-resolution, T1-weighted structural images were scanned using a 3D MDEFT (modified driven equilibrium Fourier transform) sequence (voxel = 0.5 × 0.5 × 0.5 mm^3^). Throughout the MRI session, we maintained the monkeys under anesthesia. Anesthesia was introduced with an intramuscular injection of medetomidine/midazolam (30 μg/kg and 0.3 mg/kg, respectively) and ketamine (0.5 mg/kg) before MRI scans. During acquisition of MRI, anesthesia was maintained with continuous intravenous infusion of propofol (5–10 mg/kg/h) and intramuscular injections of xylazine (1 mg/kg) as needed. Glucose-lactated Ringer’s solution was given intravenously (5 ml/kg/h). Heart rate, oxygen saturation, and blood pressure were continuously monitored.

### Recording Electrodes

**Figure 2.**
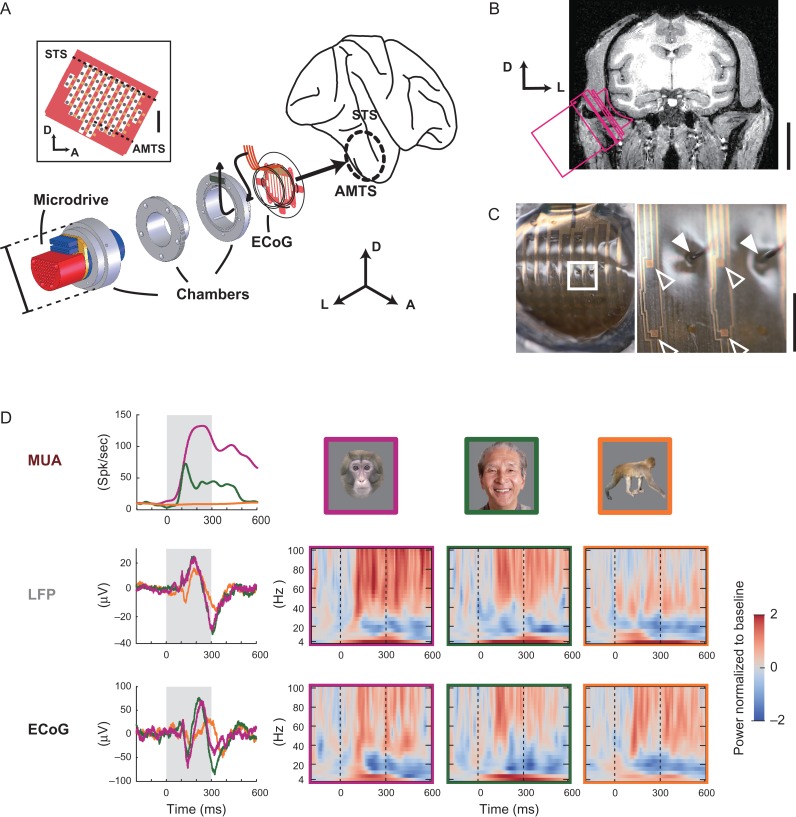
Simultaneous ECoG, MUA, and LFP recording system for the macaque ITC. (*A*) ECoG electrodes attached to the bottom of a silicone “artificial dura,” titanium chambers, and the microdrive are shown in their order of placement on the animal’s head. Also see Figure 2 and Supplementary Figure S1. (Inset) Schematic drawing of ECoG (yellow) and microelectrode (black) spatial configurations on the cortex. Electrodes were placed on area TE of the ITC, covering the IT gyrus and extending marginally below the anterior medial temporal sulcus (AMTS). The area in red corresponds to the Parylene-C insulation film, which is transparent (see photograph in *C*). (*B*) The electrode assembly accessed the IT gyrus at a position and angle pre-allocated by an anatomical MRI scan. (*C*) Surface of the electrode assembly seen from below. The region marked by a white square on the left is magnified on the right. Open arrowheads indicate ECoG contacts. Closed arrowheads indicate microelectrodes used for MUA and LFP recordings, which penetrate the slits in the Parylene-C insulation and the overlaying silicone membrane. (*D*) Representative visual responses of ECoG, LFP, and MUA from the same (adjacent) recording site. ECoG and LFP responses are shown in average waveforms (left) and normalized power spectrograms (right). MUA responses are converted to spike density functions (Materials and Methods). Top right are stimulus images; colored frames do not appear as part of the stimulus but represent a correspondence to the response waveforms and the response spectrograms. Shaded areas behind the waveforms and vertical dotted lines on the spectrograms show the stimulus presentation periods. Scale bars, 25.4 mm (A), 5 mm (A inset), 20 mm (B), and 1 mm (C right).

The multimicroelectrode array used for MUA and LFP recording was customized from a commercially available semichronic microdrive system (SC60-1; Gray Matter Research, Bozeman, USA). The array consisted of 60 microelectrodes arranged in a grid configuration with 1.2-mm interelectrode spacing (Fig. 2*A*). Each microelectrode was 75-μm-diameter iridium coated with Parylene-C (Poly(chloro-*para*-xylylene)) having typical impedance of 0.5 MΩ measured at 1 kHz. ECoG electrodes were prepared via micromachine techniques using 0.25-μm-thick gold wiring and 10-μm-thick Parylene-C insulation with the recording contacts exposed in a 100 × 100 μm square shape (Fig. 2*C*, Supplementary Fig. S1B). ECoG contacts were arranged in a grid shape matching the spatial configuration of the multimicroelectrode array (Fig. 2*A* inset, Fig. 2*C*). The lead wires and Parylene-C insulation were aligned in columns with slits between them (Fig. 2*C*, Supplementary Fig. S1B). A pair of cable bundles led from the ECoG probe to two 0.025-inch pitch 36-pin connectors (Supplementary Fig. S1B, C; #A8828-001-vv; Omnetics, MN, USA). Additional details on the ECoG-manufacturing process have been described previously (Takeuchi et al. 2005; Toda et al. 2011). Gold–Parylene-C ECoG electrodes were attached to the bottom of a silicone artificial dura (Fig. 2*A*), which resembled the design of the “artificial dura” used in *in vivo* optical imaging techniques (Arieli et al. 2002). Small protrusions of the insulation film were inserted into the slits on the brim of the artificial dura and fixed using a small amount of silastic rubber for mechanical stability (Fig. 2*A*). ECoG probe and microelectrode array were assembled together and implanted onto the cortical surface on area TE of the IT cortex (Fig. 2*A*, *B*).

### General Surgical Procedures

General procedures of the surgery largely overlap with those described in a previous report (Matsuo et al. 2011). Anesthesia was introduced with an intramuscular injection of medetomidine (30 μg/kg) and ketamine (1 mg/kg). Animals were artificially respirated with oxygen and maintained in anesthesia with isoflurane (1–2%) during the surgeries. The venous line was secured using lactated Ringer’s solution, and ceftriaxone (100 mg/kg) was dripped as a prophylactic antibiotic. Animals received ketoprofen as an analgesic for 3 days, and the antibiotics were continued for 1 week after surgery. Oxygen saturation, heart rate, and end-tidal CO_2_ were continuously monitored (Surgi Vet; Smiths Medical PM Inc., London, UK) throughout surgery to adjust the levels of anesthesia. Body temperature was maintained at 37 °C using an electric heating mat. The skull was fixed with a 3-point fastening device (Integra Co., NJ, USA) with a custom-downsized attachment for macaques and a vacuum-fixing bed (Vacuform, B.u.W.Schmidt GmbH, Garbsen, Germany) was used to maintain the position of the body. Following skin incision, zygomatic arch, temporal muscle, and the upper portion of the mandible bone were removed to facilitate the approach. A burr hole was opened in the inferior temporal portion of the skull (Fig. 2*B*) by a perforator (Primado PD-PER; NSK, Tochigi, Japan) with an attachment for infants (DGR-OS Mini 8/5 mm R; Acura-Cut Inc., MA, USA). Hemorrhage from the dura was controlled by a bipolar coagulator (Bipolar SX-2001; Tagawa Electronic Research Institute, Chiba, Japan).

### Implant Surgery

We implanted the chronic recording device from the temporal side (Fig. 2*A*, *B*). An artificial dura that has the ECoG probe attached to its bottom (Fig. 2*A*, Supplementary Fig. S1A, D) was placed onto the surface on area TE of the IT cortex, covering the IT gyrus and extending slightly below AMTS (Fig. 2*A*, *B*), through a window on the dura. A 3-piece metal chamber system was used as the interface between the skull and electrode arrays. The bottom chamber (Fig. 2*A*, Supplementary Fig. S1A, C) fit tightly to the craniotomy window that was made on the skull. Titanium anchor screws were placed on the skull, and dental resin firmly attached the chamber to the skull. Canals on the inner wall of the chamber and the protruding ridges on the outer wall of the cylindrical part of the artificial dura aligned the ECoG probe and the microelectrode array. The middle chamber was slowly inserted into the inner wall of the cylindrical part of the artificial dura (Fig. 2*A*, Supplementary Fig. S1A, D), whereas the wall of the artificial dura was securely held up with a 5-0 nylon thread. ECoG lead wires exited through an opening located between the 2 chambers (curved arrows on the ECoG probe in Fig. 2*A*, Supplementary Fig. S1D), and the 2 chambers were firmly attached by screws. The opening made for the ECoG wire was later closed with a quick-curing silastic rubber (kwik-sil; WPI, Sarasota, USA). The microdrive was inserted into the second chamber, and the third piece of the chamber was firmly screwed to the second piece, thereby attaching the microdrive to the second chamber. The microdrive and second chamber were precisely aligned by a pin located on the microdrive and a hole located on the second chamber. The electrode assembly accessed the IT cortex at a pre-allocated position and angle, which were determined via an anatomical MRI scan (Fig. 2*B*). The sharp iridium microelectrodes used for MU and LFP recordings penetrated through the silicone membrane and went through the slit in the Parylene-C insulation (Fig. 2*C*). ECoG contacts and microelectrodes were arranged in the same spacing and configuration but shifted by half of the spacing distance. Electrodes were placed on area TE of the IT cortex, covering the IT gyrus and extending slightly below AMTS (Fig. 2*A*, *B*).

### Daily Recordings

Daily recording experiments included 2 steps. First, the animal’s head was fixed in the chair and the quality of multi-unit recording from the microelectrodes was quickly examined qualitatively on the basis of the signal-to-noise ratio (S/N) of the signal. We adjusted the depth of the electrodes that had poor recording quality. However, to minimize the working time of the animal and the risk of pushing down the cortex, we adopted the following strategy when choosing the electrodes that were to be manipulated. In the initial 2 weeks of the experiment, up to 15 electrodes were manipulated per day. In later sessions, we took the history of recording quality into account; electrodes with a poor S/N history were left untouched, and electrodes with an intermediate S/N history were adjusted, persuading only to the level matching that of the preceding recording sessions. This allowed us to limit the electrode adjustment time to 1–1.5 h per day.

### Stimulus Image Set

The stimulus set consisted of images that belonged to 1 of 3 discrete “coarse” categories (face, body, and inanimate object) and 2 additional categories, namely modified face (parts-scrambled face and face part) and body part (hand) (Fig. 1*B*). Images that belonged to the face category were further divided into subcategories (Fig. 1*C*) that overlapped partially. One was the “species” category, which consisted of the human face group and the macaque face group. Another was the “view” category, which consisted of human face images with 3 different views, with each view having 5 different identities. The same image set was also used as the “identities” category, which was set up by grouping the images into different identities, with each identity having 3 different views.

### Data Analysis Part 1: Data Acquisition, Frequency Spectrum

#### Data Acquisition

MUA, LFP, and ECoG data were simultaneously recorded using the TDT System3. MUA and LFP were recorded from the 60 penetrating microelectrodes, and ECoG was recorded from the 60 surface-contact electrodes. Signals were fed to headstage amplifiers (ZC 32 and ZC64, TDT) and a preamplifier/digitizer (PZ2, TDT) and then fed into the digital signal-processing module (RZ2, TDT). For multi-unit data, the signal was band-pass filtered between 300 Hz and 5 kHz, and the time points at which the waveform exceeded 3.7 × the standard deviation (SD) of the signal were stored as multi-unit time stamps. For LFP and ECoG data, the signal was initially stored in wide band (no digital filtering). Acquired data were analyzed with in-house programs that run on MATLAB. Visually evoked MUA was converted to spike density function using kernel optimized for the spiking rate of each of the respective stimulus condition (Shimazaki and Shinomoto, 2010; Fig. 2*D*). A multi-unit was considered to be visually responsive if the firing rate in the visual stimulation period and that in the prestimulus period differed with statistical significance (*P* < 0.05, 2-sample Kolmogorov–Smirnov test, corrected for multiple comparisons using the Bonferroni method by the number of stimuli).

#### Features of MUA

We used the frequencies of spiking activity of MUA as input features for classification. Unless stated otherwise, spike rates from multiple electrodes and time windows were combined. We used MUA signals during a period from −50 ms to 600 ms relative to the stimulus onset in each trial. The signal at each microelectrode was sampled using a 100-ms time window that was shifted by 50 ms, and the spike rate in each time window was calculated. The spike rates of all microelectrodes and the 12 consecutive time windows were used as the input features to a decoder. The features used for characterizing the time course of decoding accuracy were limited to a single time window. The spike rates of all electrodes in a single 100-ms sliding time window were used. The time window was slid by 25 ms, and the decoding accuracy was calculated as a function of time. The spike rates of a single electrode from the 12 time windows were used for characterizing the decoding accuracy of each single electrode. We excluded the MUA data that did not yield significant visually evoked response, as defined by pair-wise Kolmogorov–Smirnov (*P* < 0.05, corrected by Bonferroni method with the number of stimulus images) between the prestimulus period and the evoked period.

#### Features of ECoG and LFP Signals

**Figure 3.**
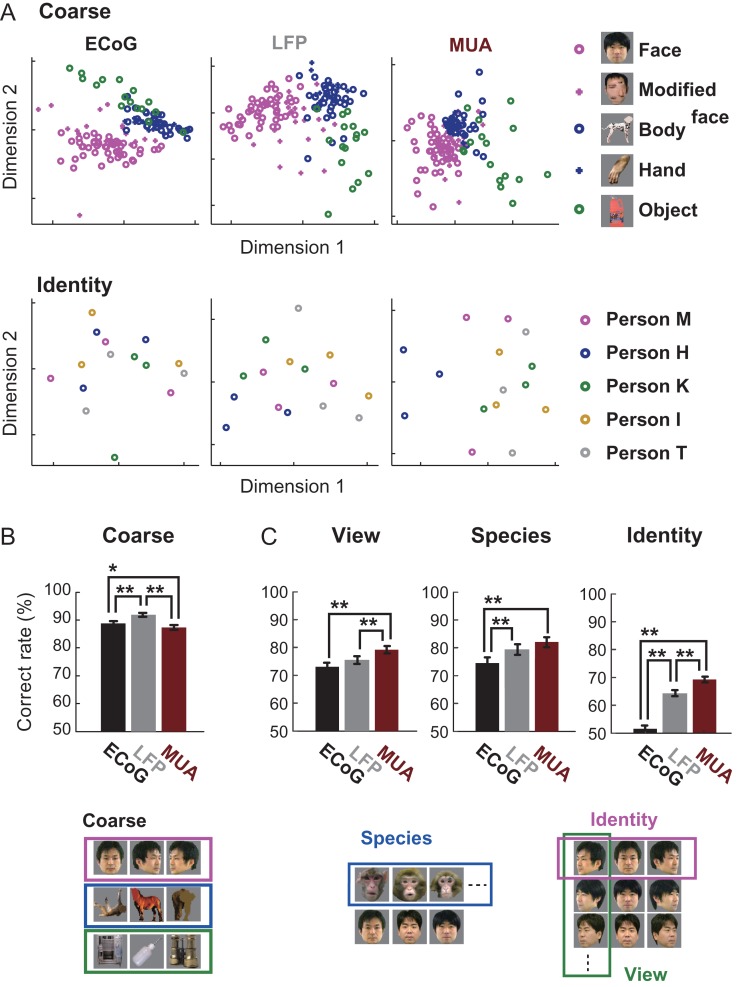
Category representation by the IT neural response recorded using different measurement methods. Feature vectors were total power for ECoG and LFP as well as the average spike rate for MUA, calculated in the same time windows (Materials and Methods). (*A*) Two-dimensional MDS plot of the coarse category (face, modified face, body, body parts, and inanimate objects) response vectors from monkey H in ECoG, LFP, and MUA response spaces. (*B*) Coarse category (face, body, and inanimate object) decoding performance using linear SVM for the respective measurement methods pooled from 2 animals. The number and position of the channels were balanced between measurement methods by subselecting ECoG channels at the sites of intact microelectrode channels. Chance level was fixed to 50% by randomly subselecting the training data for an equal number of stimuli in class and non-class (see Materials and Methods for details). (*C*) Comparison of fine category decoding performance between ECoG, LFP, and MUA responses. Species data were pooled from 2 animals. View and identity data were from monkey H. *P < 0.05; **P < 0.01. Comparisons were made by a chi-squared test with Bonferroni correction principle for multiple comparisons.

For classification, we used the mean amplitudes and spectral powers of the ECoG/LFP signals as input features. To compare the decoding performance with that obtained using MUA, we excluded the data from the ECoG (LFP) electrodes that overlay (matched) the microelectrodes that did not yield good MUA signals. We used ECoG/LFP signals during a period from −50 ms to 600 ms relative to the stimulus onset in each trial. Unless stated otherwise, the mean amplitudes and spectral powers from multiple electrodes and time windows were combined. Two types of features were computed from ECoG/LFP signals: one was the total power summed across the frequency spectrum, while the other was the wavelet power separately obtained for respective frequencies. To obtain the total power, the signal at each electrode was sampled using a 100-ms time window that was shifted by 50 ms, and the spectral powers of the 101 frequency bands (10–1000 Hz, with 10-Hz intervals) in each time window were calculated using Fast Fourier Transform. The mean of all the frequency powers was taken as the “total power” of the time window, and the total powers from all electrodes and the 12 consecutive time windows were used as the features of input into a decoder (Fig. 3*B*, *C*). To obtain the wavelet power, the original signal was convoluted with a Gabor (Morlet) wavelet, with the sinusoidal carrier frequencies in theta (4 Hz), alpha (12 Hz), beta (24 Hz), low gamma (40 Hz), and high gamma (80 Hz). DC was the mean of the squared raw voltage values within the time window. The wavelet at each frequency had a Gaussian envelope width (σ) that was equal to the cycle period (frequency^−1^) of the carrier and had tail truncation at 2σ of the Gaussian envelope (double of the carrier cycle period). The spectrograms obtained after the power of each frequency was normalized to the power observed in the prestimulus period (−200 to 0 ms) are shown in Figure 2*D*. The mean of the total power from the time bins in the range of 50–450 ms was used in multidimensional scaling (MDS) analysis (Fig. 3*A*). In the analysis performed to compare stimulus selectivity and decoding accuracy between frequency bands, the power of each frequency was binned within the 100-ms time window that was shifted by 50 ms. For stimulus selectivity analysis (Supplementary Fig. S2) and for generating the category selectivity d′ map (Fig. 5), the response of the respective frequency band was the mean of time bins in the 50–450-ms range, collected for each channel. Stimulus selectivity was compared between the trial-averaged data of respective measurement methods. The d′ map was generated using the mean of odd trials to compute the preferred category and using the even trials to compute the d′ of the preferred category.

**Figure 4.**
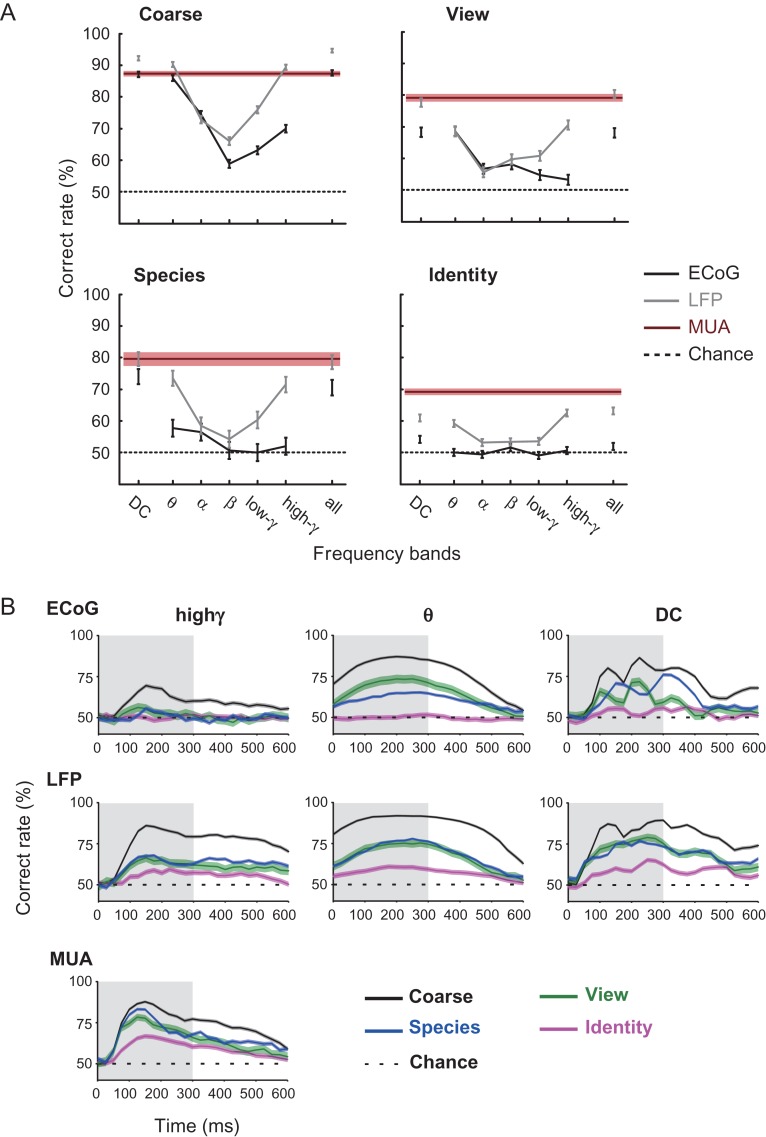
Recording method- and frequency-dependent category decoding performance. (*A*) Feature vectors were raw event-related potential (displayed as DC) and respective wavelet frequency powers (Materials and Methods). Decoding performances were separately computed for coarse, view, species, and identity categories for the respective frequencies. Note that the number of features before feature selection was identical across methods and frequencies, with the exception of the “all” condition. An equal number of features were selected across frequency bands, including the “all” condition. Each line color represents the performance of each recording method, which is denoted in the caption. Error bars and red shadings around the MUA lines indicate the 95% confidence limit, assuming binomial distribution. (*B*) Time course of category decoding performance across recording methods (ECoG, LFP, and MUA) and frequency bands (high-gamma, theta, and DC bands). Each colored line represents performance in each category denoted in the caption. The details of the feature extraction and the decoding methods were equivalent to those described in Figure 3, with the exception that features from the corresponding time bins were used at each time point (Materials and Methods). Shadings show the stimulus presentation period.

For frequency-dependent decoding analysis (Fig. 4*A*), power from all electrodes and the 12 consecutive time windows for the respective frequencies was taken as the features of input into a decoder. The features used for characterizing the time course of decoding accuracy were limited to a single time window (Figs. 4*B* and 6*A*, B). The mean amplitudes and powers of all electrodes from a single 100-ms sliding time window that was slid by 25 ms were used, and the decoding accuracy was calculated as a function of time. Phase-locking value (PLV) of the theta frequency was computed from the theta wavelet phase response (Fig. 6*C*). First, the phase of each channel at a fixed post-stimulus time point was plotted as unit-length vectors in the complex plane. Then, the PLV was computed as the length of a vector-sum (resultant vector) of these channel-wise theta phase vectors in the complex plane. Statistically significant difference of the PLV values was evaluated by Mann–Whitney *U*-test for species (human/monkey) categorization and by Kruskal–Wallis test for facial view (right/center/left) categorization. Pair-wise difference between the facial views was tested with *post-hoc* Bonferroni–Dunn method. The fixed post-stimulus time point was set to 75 ms after the stimulus onset, where difference between the LFP and ECoG time course reached its maximum slope.

### Data Analysis Part 2: Decoding

#### Decoding Analysis

Using a neural decoding approach, the efficacy of extraction of visual object information from single-trial signals was compared between ECoG, LFP, and MUA. The decoding performance of each signal method was evaluated by pair-wise decoding analysis. We selected a pair of object categories and selected the trials in which the images included in those 2 categories were presented. Using those trials, a binary classifier (decoder) was trained to predict the category of a presented image on a trial-by-trial basis and was tested (Kamitani and Tong 2005). We applied this procedure to all pairs of the 3 coarse categories (face, body, and inanimate object); modified face and body part were not included in the decoding analysis because they do not fully qualify as the face or body category. All pairs of the 3 view categories, all pairs of the 5 identity categories, and the pair of the 2 species categories were decoded similarly. Each binary decoder consisted of a linear support vector machine (Vapnik 1998) implemented by LIBSVM (Chang and Lin 2011). Before decoder training, we used a feature-normalization procedure and a feature-selection procedure. In the feature-normalization procedure, the values of each feature were z-transformed using the sample mean and SD calculated using the training data set. In the feature-selection procedure, the dimensionality of the feature vector was reduced by selecting informative features on the basis of univariate analysis (*F*-statistics) applied to the training data set. We ranked the features according to the *F*-value that indicated differential responses to the categories, and the top 100 features were used as input into the decoder. In cases in which the number of original features used for classification was equal to or less than 100, we omitted this feature-selection procedure and used all features. Decoding performance was evaluated by cross-validation analysis. To evaluate generalization performance for category classification across different exemplars, we ensured that trials that corresponded to the same visual stimuli were not included in the training and test data sets (Vindiola and Wolmetz 2011). For each category pair, we randomly selected *N* exemplars per category. *N* was set to the number of the exemplars of the category that had fewer exemplars than the paired category. We divided the *N* × 2 exemplars into *N* groups, each of which contained 2 exemplars from the 2 different categories and divided the corresponding trials into *N* groups. (*N* − 1) groups were then used to train a decoder, and the remaining group was used to evaluate the trained decoder. This procedure was repeated until the trials from all *N* groups were tested (*N*-fold cross-validation), and the percentage of correct classification was calculated.

#### Decoding with Spatial Shuffling

For spatial shuffling, we shuffled the original wavelet power response vectors (ECoG and LFP) or the spike rate response vectors (MUA) in the spatial domain by exchanging the channel label for each stimulus presentation trial. The range of spatial shuffling varied from 4 to 60 channels (Supplementary Fig. S5B). We quantified the drop in decoding performance on the basis of the difference in performance in the condition without shuffle and the condition with a maximum 60-channel shuffle (Supplementary Fig. S5). The maximum drop in decoding performance and the drop rate (sharpness of the drop) were quantified in the same manner as that in the spatial shuffling. We also quantified the drop rate of decoding performance with regard to shuffling. The decoding performance was fit with a curve that was defined as *y* = A exp(−B*x*) + C (Supplementary Fig. S5A; *x*, size of the subarea used for shuffling; *y*, classification performance; A, B, and C, constants [A > 0, B > 0, and C > 50], B is the decay constant), using Matlab Curve-Fitting Toolbox.

#### Decoding with Trial Shuffling

To characterize the effect of correlations among channels, we performed the decoding analysis with trial-shuffled data. See Majima et al. for a detailed explanation on the effect of trial shuffling to multichannel field potential data (Majima et al. 2014). For category decoding with shuffled training data and original test data, training data were shuffled across the trial for every *N*-fold cross-validation procedure. For category decoding with shuffled training and test data, the original data were first shuffled across trial and processed for further decoding analyses.

## Results

To explore and compare spatiotemporal organizations for ordinate and subordinate categories in the ITC, we recorded neural activity from 2 monkeys (Macaca fuscata) performing a passive viewing task. In this task, the animal must maintain fixation while 2 or 3 visual stimuli from a hierarchically categorized stimulus set were sequentially presented (Fig. 1*A*). Visual stimuli were classified into 3 “coarse (ordinate)” categories (face, body, and inanimate object; Fig. 1*B*), and the face category was divided into subordinate categories (Fig. 1*C*) based on “species” (human faces and macaque faces). Human face category was further divided into “view” (3 different views of human faces) and “identity” (5 individuals regardless of the view angles) subcategories.

Our novel electrode assembly enabled simultaneous high-density recording of MUA, LFP, and ECoG from a 12 mm × 12 mm local region in the anterior ITC (Fig. 2*A*, *B*). MUAs (Fig. 2*D* top left) and LFPs (Fig. 2*D* middle) were recorded from the same penetrating microelectrode array (Fig. 2*C* closed arrowheads; see black spots in Fig. 2*A* inset for the spatial arrangement). ECoG (Fig. 2*D* bottom) was recorded from the surface electrode array (Fig. 2*C* open arrowheads; see yellow spots in Fig. 2*A* inset for the spatial arrangement) that covered the same local cortical region. The microelectrodes penetrated the slits in the ECoG probe, avoiding electrode contacts and lead wires (Fig. 2*C*).

### Spatiotemporal Homogeneity of Category-Encoding Neural Activity Depends on the Ordinate Level of the Category

We compared the amount of category information obtained from the multichannel patterns of visually evoked MUA, LFP, and ECoG signals that record neural activity with different scales of spatial and temporal summation. MDS and decoding-based analyses were performed by extracting the same number of features from the respective recorded data sets: total powers from ECoG and LFP and mean firing rate from MUA (see Materials and Methods). MDS revealed that with all the 3 recording methods the visual responses to coarse categories (faces, bodies, and inanimate objects) showed a clear tendency to form discrete clusters (Fig. 3*A*). To estimate the spatiotemporal scale and homogeneity of functional neuronal clusters representing multiple levels of visual category, we examined how reliably the stimulus category was decoded from single-trial ECoG, LFPs, or MUA using a linear support vector machine (Vapnik 1998). The generalization accuracy for the coarse category classification (Fig. 3*B*) was well above the chance level of 50% for all 3 recording modalities (see Materials and Methods). In particular, the single-trial ECoG and LFPs carried sufficient information for predicting the coarse category with a correct classification rate of 88.9% and 92.0%, respectively. These were significantly higher (*P <* 0.05 and *P <* 0.001, chi-squared test corrected for multiple comparisons) than the performance obtained using MUA responses (87.4%), indicating that summation of neural activity in a certain spatiotemporal scale enhanced the coarse category selectivity. However, for subordinate category classifications, MUA was the best of the 3 recording methods (Fig. 3*C*, brown bars) – MUA (69.2%) and LFP (64.3%) carried significant facial identity information, whereas ECoG (51.5%) did not (Fig. 3*C* right). The correct classification rates were 79.2% (MUA), 75.5% (LFP), and 73.0% (ECoG) for facial view angles (Fig. 3*C* left), and 82.2% (MUA), 79.5% (LFP), and 74.6% (ECoG) for facial species (Fig. 3*C* middle).

The superiority of MUA suggests that subordinate categories are encoded in finer and/or more heterogeneous spatiotemporal patterns. For example, the activity of neighboring neurons may be tuned to different individuals (identity), where they could be considered similar in a sense that both are tuned to the face category. Otherwise, population neuronal responses selective to facial identities may be temporally incoherent. In any case, columnar or larger scale spatiotemporal summation of neuronal activity may result in substantial reduction of the subordinate category information, whereas the coarser category information was relatively preserved or enhanced. Decoding of the species and the view categories had characteristics that (1) differed from the coarse category decoding in that performance with MUA was superior to ECoG and (2) differed from the identity decoding in that ECoG showed moderately but significantly above-chance decoding performance. Because these 2 categories considerably have intermediately fine and/or homogeneously patterned cortical representations, we call them “intermediate categories” from here on.

### High-Frequency LFPs Specifically Contains Spike-Coupled Category Information

In the analyses so far described (Fig. 3), category decoders used total power of ECoG and LFP discarding frequency-specific features for comparison of the detectability by LFP, ECoG, and MUA with an equal number of features. However, it is plausible that powers in different frequency ranges carry qualitatively independent information having affinity to distinct types of the source neural signal (e.g., either input- or output-related signal of the recorded cortical region). Here, we tested a possibility that low-frequency LFPs carry spike-independent and input-biased category information whereas high-frequency LFPs carry category information tightly coupled to the output spike firing in the ITC, as has been reported for evoked visual responses in the V1 (Belitski et al. 2008). We first examined correlations of stimulus selectivity, rather than category selectivity, across the recording modalities in different frequency ranges (Supplementary Fig. S2). We found that the stimulus selectivity of theta-band (4 Hz) ECoG power strongly correlated with that of theta-band LFP (*R =* 0.81, *P =* 1.8 × 10^−35^). High-gamma-band (80 Hz) ECoG and LFP exhibited a significant *(R =* 0.38, *P =* 2.0 × 10^−6^) but weaker correlation. In contrast, MUA correlated strongly with high-gamma-band LFP (*R =* 0.61, *P =* 2.5 × 10^−16^), but not significantly with theta-band LFP (*R =* 0.029, *P =* 0.72), theta-band ECoG (*R =* 0.010, *P =* 0.90), or high-gamma-band ECoG (*R =* 0.049, *P =* 0.56). This method-specific and frequency-specific correlation, observed in 2 monkeys across channels (Supplementary Fig. S2B), indicates that LFP carried MUA-coupled stimulus information in the high-frequency powers, but not in the low-frequency powers. To address whether the method and frequency dependency found in the stimulus selectivity is also found in the category selectivity, we decoded multiple level of categories from the stimulus-evoked ECoG and LFP in each frequency range separately (Fig. 4*A*). For ECoG-based coarse category decoding, the correct classification rate was highest when low-frequency components such as DC and theta power were used (Fig. 4*A* top left, black line). In higher frequency ranges, the performance was above the chance level but was less accurate, with beta power giving the minimum performance. Although the overall frequency profile of LFP-based coarse category decoding (Fig. 4*A* top left, gray line) was similar to that of ECoG (Fig. 4*A* top left, black line), the classification rate with high-gamma LFP was notably higher than high-gamma ECoG, and comparable to the performance with theta LFP. This finding implies that the high-gamma LFP contains MUA-coupled category information, which the high-gamma ECoG does not contain. In facial identification with LFP, the maximum classification rate was obtained with high-gamma component (Fig. 4*A* bottom right, gray line), which is also consistent with the idea that high-gamma LFP carried fine category information coupled with MUA.

### Subordinate Category Decoding Depends on Recording Method and Signal Frequency

The classification levels of coarse category were similarly high regardless of whether low-frequency LFPs/ low-frequency ECoG or high-frequency LFPs/MUA were used (Fig. 4*A* top left). In contrast, the classification level of intermediate category (facial species and facial view) depended both on the spatial summation specific to the recording method and on the frequency of the signals used as features for machine learning (Fig. 4*A* top right and bottom left). Low-frequency components (e.g., theta power and DC) of LFP and ECoG both classified the intermediate categories significantly above chance. When the high-frequency component (e.g., high-gamma power) was used, however, the classification was significant with the less spatially summated LFP, but not significant with the more summated ECoG (Fig. 4*A* top right and bottom left). These results led us to a hypothesis that 1) for coarse categories, the functional architecture based on high-frequency LFPs may be similarly organized as those based on low-frequency LFPs/ECoG and that 2) for the intermediate, species and view categories, the low-frequency field signals form neural clusters with intermediate spatiotemporal homogeneity whereas the high-frequency field signals were relatively distributed or heterogeneous, forming no electrocorticographically detectable homogeneous clusters, in the macaque ITC.

### Double Dissociation of View and Species Decoding Between Early Theta ECoG and Late High-Gamma LFP

There is an interesting contrast between the temporal profile of the facial species decoding and facial view decoding. In the early “evoked” period of the visual response (100–200 ms after the stimulus onset), where the initial synaptic inputs and polysynaptic activity should dominate (Mitzdorf 1985), the correct classification rate with theta ECoG (Fig. 4*B* top center) was higher for view (green) than for species (blue). The classification rate with early high-gamma ECoG (Fig. 4*B* top left) was much lower but exhibited similar tendency. In this early evoked period, however, there was no difference between view and species decoding with theta LFP (Fig. 4*B* middle center) or high-gamma LFP (Fig. 4*B* middle left). In contrast, in the late “induced” period of the visual response (300–500 ms after the stimulus onset), species decoding with high-gamma LFP was slightly superior to view decoding (Fig. 4*B* middle left). Superiority of species decoding to view decoding was observed neither with high-gamma ECoG nor with theta LFP/ECoG. These findings suggest that category information extractable from the activity of neural clusters in the ITC not only depends on the method-specific spatial summation and the frequency of neuronal synchrony but also on the latency, namely the early “evoked” period and the late “induced” period, underscoring the necessity to scrutinize the category-specific functional architecture of early evoked theta LFP/ECoG and late-induced high-gamma LFP separately.

### Mapping Category-Selective “Homogeneous Clusters” in the Cortical Space

**Figure 5.**
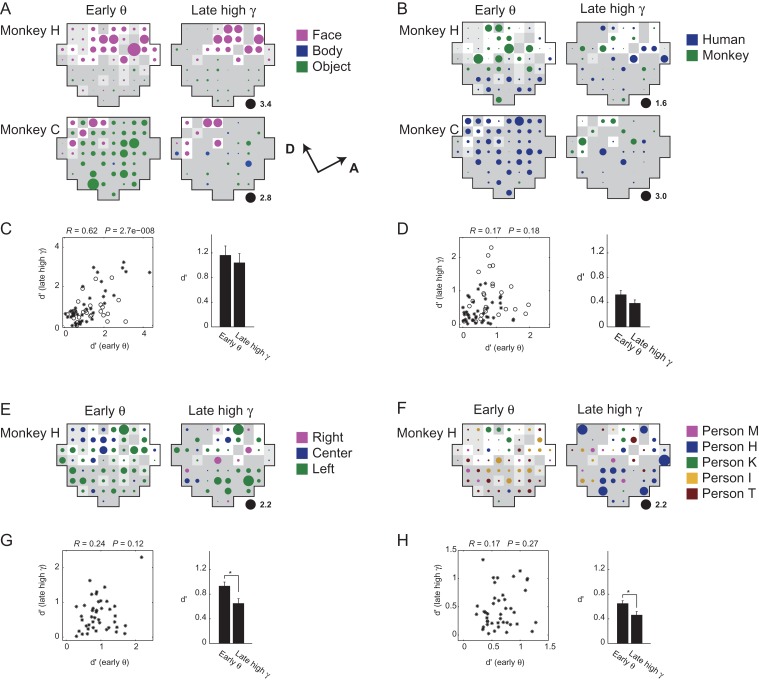
Spatial profile of category selectivity measured by LFP. (A, B, E, F) d′ category selectivity maps for coarse (*A*), species (*B*), view (E), and identity (F) categories, respectively. The maps were generated from early theta (left) and late high-gamma (right) LFP responses that had significantly large spectral power during the stimulus-evoked period. Color and size of the patches depict preferred category and the d′ magnitude. Black patches indicate scale of the d′. White background color depict region showing strong face preference in coarse categorization (d′ > 1), light gray indicates mild face preference (d′ > 0.5), dark gray otherwise (*C*, *D*, *G*, *H*). The theta and high-gamma d′ of each recording site are plotted in scattergrams (left), and mean values (right). Only the channels with significant evoked power in both the early theta and the late high-gamma signals were used. *P < 0.05. Comparisons by pair-wise *t-*test corrected for multiple comparison with Bonferroni correction. Error bars indicate the standard errors.

To test whether the category-encoding “spatiotemporally homogeneous neural clusters” implied by the decoding analyses correspond to the actual clustering of neurons with similar category selectivity in the cortical space, we examined spatial patterns of category selectivity maps (d′ maps) generated from the early low-frequency LFPs and the late high-frequency LFPs for both monkeys (Fig. 5). We found that the category-specific decoding performance with LFPs (Fig. 4) approximately corresponded to the strength of channel-wise selectivity (d′ value depicted by the diameter of colored circles in Fig. 5), which we speculate to reflect a local, columnar-scale (several hundred micrometer) summation of similar category-selective neuronal activity. In contrast, the decoding performance with ECoGs appeared to reflect a larger, across-channel (several millimeter) homogeneity of category selectivity in early low-frequency LFP maps. Typically, the coarse category maps exhibited a group of face-selective channels in the anterior part of the chamber for monkey H (Fig. 5*A* top), and in the dorsal part for monkey C (Fig. 5*A* bottom). The early theta-defined view categorization map was dominated by a large “left-view”-selective homogeneous region except for a small region in the dorsal portion within the chamber (Fig. 5*E* left). Similarly, the early theta-defined species categorization map exhibited a “monkey face” selective dorsal region for monkey H (Fig. 5*B* top left), or a larger but weakly selective “human face” region for monkey C (Fig. 5*B* bottom left). The late gamma-defined categorization maps tended to have more distributed form for both view and species categorization (Fig 5*B*, *E* left). Interestingly, the channels selective to particular facial species, facial views, and facial identity spanned not only within but also outside of the face-selective region (see light-colored region in Fig 5*B*, *E*, and *F*).

These results suggest that not only the spatial clustering but also spatially spanned homogeneity of low-frequency neuronal activity is the physiological correlate of the “spatiotemporal homogeneous clusters” implied by the decoding-based analysis.

### Spatial Factors Partially Explain Dissociation Between View and Species Decoding

**Figure 6.**
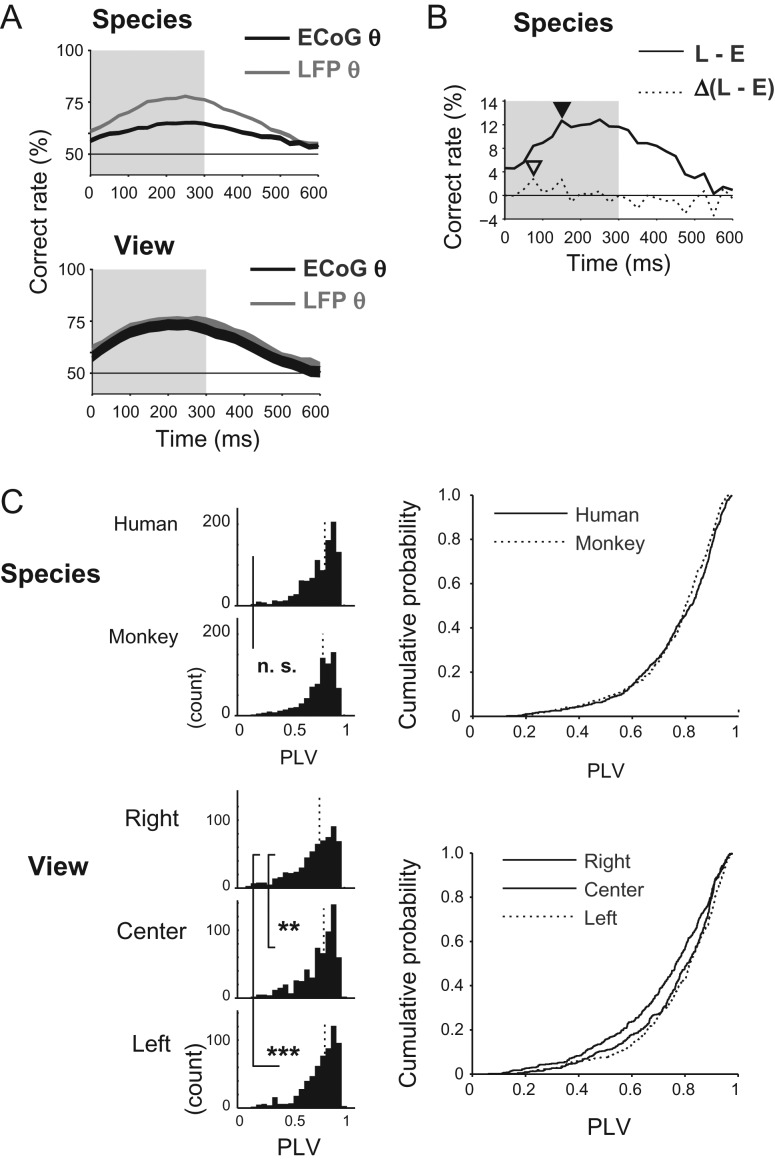
ECoG signal-specific attenuation of species category information. (*A*) Comparison of theta-band ECoG and LFP in view (top) and species (bottom) decoding time course. (*B*) Differences between LFP and ECoG decoding time course (solid line) and its temporal differential (broken line). Closed and open arrowheads indicate respective peak positions. (*C*) (left) Distribution of theta-band PLV computed across channels, sorted by view and species, and pooled across trials and recording days. (right) Cumulative probability plot of the theta PLV. **P < 0.01, ***P < 0.001, n.s. not significant. Comparisons by Wilcoxon rank-sum test (species) and Kruskal–Wallis test with *post-hoc* pair-wise comparisons using Dunn’s method corrected for multiple comparison with Bonferroni correction (view). Dotted vertical lines indicate the median. Shaded areas in gray (A and B) represent the stimulus presentation period.

Does the spatial clustering give a reasonable account on the double dissociation of the view and species decoding between the early theta ECoG and late high-gamma LFP? The left-view–selective cluster in the view early theta d′ map (Fig. 5*E* left) was larger but more heterogeneous than the human-selective cluster in the species d′ map (Fig. 5*B* left). The larger spatial span of the signal source is advantageous, but the heterogeneity of the signal source is disadvantageous for decoding with ECoG signals that go through extensive spatiotemporal summation. To quantify the net effect of larger but more heterogeneous clustering of the view-selective signals in comparison to the species-selective signals, we conducted decoding analysis using spatially shuffled LFP data (Supplementary Fig. S5), where the channel assignment within various-size subareas of the chamber was randomly shuffled (Materials and Methods; Supplementary Fig. S4A). As the shuffled area size increased, the early theta LFP-based decoding performance decreased more gradually for view than species, which was exemplified by the smaller spatial decay constant (Fig. S4B inset). The results indicate that the positive effect of the larger cluster size overrode the negative effect of its heterogeneity, which may explain why loss of the decoding performance with the early theta ECoG compared with the early theta LFP was milder for view than species categories (Fig. 4*B* top center, Fig. 6*A*).

In the post-stimulus–induced period (after 300 ms), decoding performance with high-gamma LFP was higher for species than for view (Fig. 4*B* middle left), whereas no species or view information was detectable in high-gamma ECoG (Fig. 4*B* top left). The spatial shuffle analysis confirms that the late high-gamma LFP-based decoding was more robust for species than view, as indicated by the smaller spatial decay constant (Fig. S4B). These results are consistent with the late high-gamma d′ maps showing a more mosaic-like distribution for view-selective channels than species-selective channels (Fig. 5*B*, *E*).

### Temporal Factors Contributing to Category-Selective Functional Neural Clusters

We next evaluated the possibility that reasons other than the spatial clustering, particularly temporal synchrony of neuronal population, may also significantly contribute to the formation of spatiotemporally homogeneous functional cluster sensitive to decoding. To test this possibility, we analyzed phase-locking of the evoked low-frequency LFP signals across channel, which may reflect synchrony of the inputs in the recorded region (Fig. 6*C*). The phase of the evoked theta LFP was investigated at 75 ms after the stimulus onset, where the time derivative of the differential between LFP and ECoG decoding performance reached a maximum (Fig. 6*B*). The PLV (see Materials and Methods) were significantly different across the view category members (right/center/left; *P =* 4.2 × 10^−7^, Kruskall–Wallis test), specifically between the right and center views (*P =* 0.0015, *post-hoc* Bonferroni–Dunn test) and between the right and left views (*P =* 2.9 × 10^−7^), but not between the center and left views (*P =* 0.19). The phase variability was not significantly different across species category members (human/monkey; *P =* 0.069, Wilcoxon test). These findings suggest that temporal synchrony was another significant factor contributing to the higher decoding accuracy for view compared with species using the early theta ECoG.

### Facial Subcategory-Specific Alteration of Categorical Architectures in the ITC

For the coarse category level, the face-selective domains in the early low-frequency d′ map and the late high-frequency d′ map overlapped (Fig. 5*A*) showing significant correlation (*R* = 0.62, *P* = 2.7 × 10^−8^; Fig. 5*C*), supporting the hypothesis (1) that the functional IT architecture for coarse category based on the high-frequency LFPs is similarly organized as those based on the low-frequency LFPs/ECoG. For the intermediate (facial species and view) categories, the d′ category selectivity maps defined by the early theta LFP and those defined by the late high-gamma LFP were distinct (Fig. 5*B*, *E*). Neither the species (*R =* 0.17, *P =* 0.18; Fig. 5*D*) nor the view (*R =* 0.24, *P =* 0.12; Fig. 5*G*) categories indicated significant correlation between the early and the late d′ values.

In the d′ maps of the early theta LFP, there was recognizable spatial homogeneity (Fig. 5*B* left and Fig. 5*E* left). In contrast, the d′ map of the late high-gamma LFP was more spatially heterogeneous (Fig. 5*B* right and Fig. 5*E* right). Specifically, species maps exhibited clusters both smaller in size and weak in selectivity (illustrated by small patches), indicating local mixture of neuronal activity selective to distinct species (Fig. 5*B* right). To quantify this alteration of category selectivity maps, we counted the number of category-selective channels in the early theta and the late high-gamma d′ map. The channels were considered category-selective if |d′|>1. For monkey C, human-selective channels dominated in the early theta d′ map (monkey/human = 0/29), but the dominance declined significantly in the late high-gamma d′ map (monkey/human = 5/3, *P* = 0.00013, Fisher’s exact test). For monkey H, on the other hand, monkey-selective channels dominated in the early theta d′ map (monkey/human = 6/0). The dominance also tended to decline, although this change did not reach statistical significance (monkey/human = 1/2, *P =* 0.083). The facial view map and the facial identity map exhibited mosaic-like distribution of channels selective to different views (Fig. 5*E* right) and to different identities (Fig. 5*F*), indicating extensive heterogeneity of category selectivity.

These results were consistent with the hypothesis (2) that for the intermediate categories, the low-frequency field signals are intermediately clustered and/or spatiotemporally homogeneous, whereas the high-frequency field signals were relatively distributed and/or heterogeneous. The finding that the intermediate category maps with the late high-gamma LFP did not contain highly homogeneous clusters may explain why ECoG-based decoding with a large-scale spatial summation was disadvantageous with late high-gamma signals.

## Discussion

**Figure 7.**
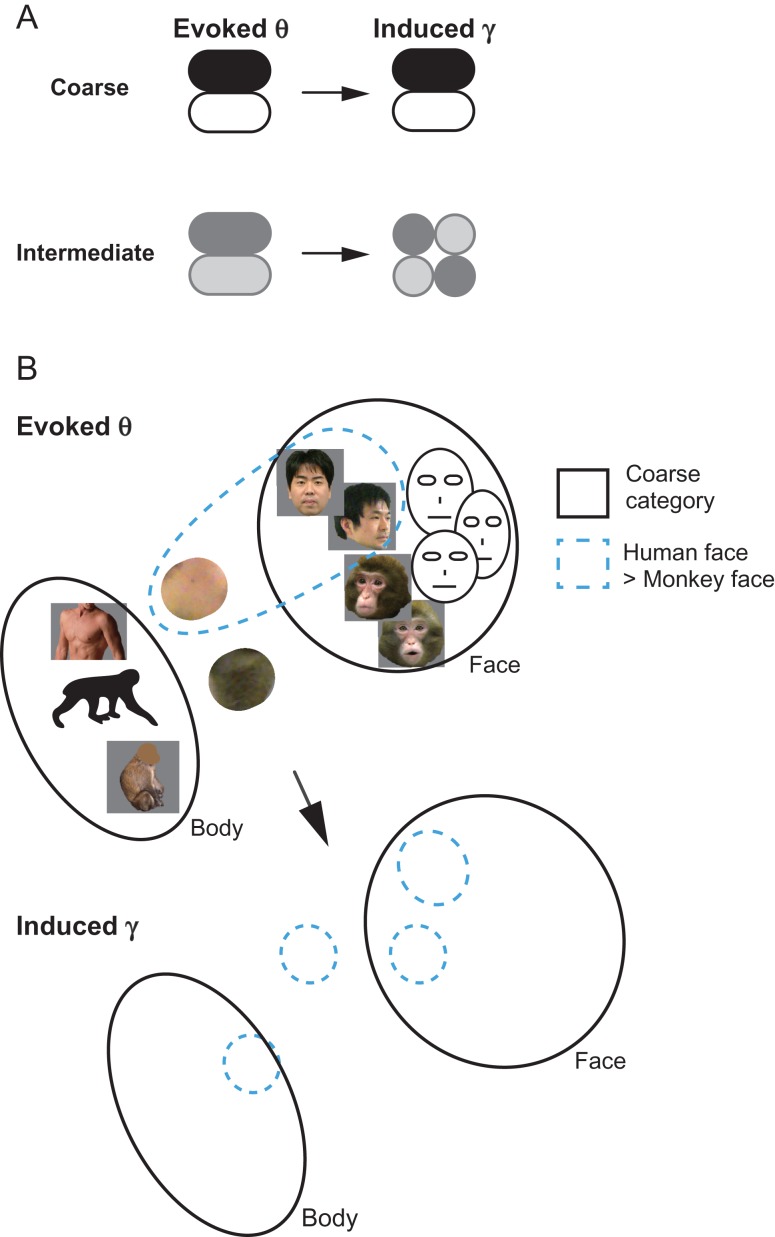
Schema showing transformation of category-selective functional maps in area TE. (*A*) Stability and homogeneity of coarse- (upper) and intermediate- (lower) level category-selective clusters in the evoked and induced period as defined with the theta and gamma activity, respectively. Dark and bright patches depict clusters for distinct categories. Contrast of the patches represents degree of category selectivity. The coarse category-encoding cluster is homogeneous and is stable across evoked and induced period. The intermediate category-encoding cluster is homogeneous with weaker category selectivity in the evoked period but becomes more heterogeneous over time. (*B*) Evoked theta (upper) and induced gamma (lower) maps showing topological relationships between the coarse category clusters (bounded by black lines) and the intermediate species category clusters (bounded by cyan dotted lines; only the human face category is depicted for clarity) in TE. Face-selective area have neurons responding to face of specific species (human or monkey face) or to face irrespective of species (depicted by face illustrations). Neurons preferring hairless skin-like texture over haired fur-like texture can help differentiate human and monkey. Not only neurons preferring face of a particular species but also combination of species-nonspecific face (face illustrations) and skin/fur texture can differentiate human from monkey, or vice versa. Note that fur/skin were not used as visual stimuli in the present study but are shown here to indicate potential nonfacial cues to discriminate between monkey faces and human faces.

In the present study, we developed a method for estimating the spatiotemporal clustering of neural activity by decoding simultaneously acquired MUA, LFP, and ECoG data. The results revealed that neuronal signals selective to the facial view and species categories formed intermediately homogeneous spatiotemporal clusters in the ITC, whereas signals selective to the facial identity category did not form clear spatiotemporal cluster. The category information extractable from LFP and ECoG data depended on the temporal frequency of the neural synchrony and changed over time between the early “evoked” period and the late “induced” period. Specifically, low-frequency evoked LFP and ECoG data contained correlated and spike-independent category information, whereas the high-frequency–induced LFP data carried information that was tightly coupled to spike firing. Importantly, in contrast to coarse category maps, which had highly homogeneous clusters that were robust across early low-frequency signals and the late high-frequency signals, the facial view and species category maps dynamically changed from moderately homogeneous organization in early low-frequency signals to more heterogeneous and distributed organization in late high-frequency signals (see Figure 7 for schemas).

Face is a core category most frequently used for assessing the categorical organization of the pattern/object vision system (what pathway) in the macaque IT cortex. Thus, although the main findings of the present study are primarily on the categorical architecture of the face category and its subcategories, we believe that our conclusions provide significant insights into the neural principle representing natural hierarchical object categories in the macaque IT cortex. These findings suggest that the category-level–dependent functional organization of spike-coupled high-gamma signals is shaped through local cortical circuits within the ITC.

### Distributed Neural Organization for Perceptually Hierarchical Categories

The visual stimuli in the current study were hierarchically structured so that faces of 5 individuals comprised the coarser “human face” category, and human faces and macaque faces comprised the coarsest “face” category. Here, we consider 2 potential models of the topological relationship between the face-selective neuronal cluster and the facial subcategory-selective neurons in the ITC. First, a “hierarchical representation model,” a natural extension of the taxonomy of perceptual categories, assumes that the ordinate-level face category-selective neural cluster is a linear sum of the facial subcategory-selective neurons. In other words, facial subcategory-selective neurons are subpopulation of the parent face-encoding cluster. An alternative “distributed representation model” assumes nonlinear relationship between the parent category and its subcategories, indicating that the facial subcategory-selective neurons are distributed outside as well as inside the face-selective neuronal cluster. Comparison of Figure 5*A*, *B*, *E*, and *F* reveals that the facial subcategory-encoding sites (human face-selective sites or left-view-selective sites) were not subpopulations of the face-selective region. For example, a group of left-view-selective sites in the d′ map with early theta signal was found in the posteroventral region within the chamber (Fig. 5*E* left), located outside the face-selective cluster (Fig. 5*A* top left). Sites selective to monkey faces partially overlapped with the face-selective cluster, but the peak position showed a posteroventral shift (Fig 5*B* top left). Likewise, some identity-coding sites (Fig. 5*F*) located outside the parent human face-selective cluster, particularly in the late high-gamma maps. Quantitative analyses shown in Figure 5*C*, *D*, *G*, and *H* and Supplementary Figure S3 show no significant correlations between the face category selectivity and facial subcategory selectivity except for facial view selectivity defined with early theta signals in a monkey. Taken together, our findings do not provide support for the hierarchical representation model, but for the distributed representation model. As the recording chamber was placed above the posterior end of the anterior middle temporal sulcus with the center of the chamber approximately 15 mm (monkey C) and 18 mm (monkey H) anterior in Horsley–Clark stereotaxic coordinates, the face-responsive area in our study likely corresponded to the “AL face patch” (Tsao et al. 2008) and the “face-domain” (Sato et al. 2013). Indeed, in the coarse category d′ maps obtained with MUA and LFP recording, the face-selective sites spanned over several millimeters on the cortical surface (Fig. 5*A*), consistent with previous descriptions (Tsao et al. 2008; Sato et al. 2013). The present results suggest that in addition to the mirror-symmetric representation of side-view faces reported by Tsao et al., distributed representation outside the AL face patch may encode facial view information. Similarly, additional information from a region outside the AL face patch may encode the species of the target face (Fig. 7*B*), as suggested by a previous report (Sato et al. 2013). Tsunoda et al. previously suggested a nonadditive relationship between neural representations of an object and representations of its parts in the macaque ITC (Tsunoda et al. 2001). From these findings, it is reasonable to suggest that such distributed and nonlinear representation may be a general rule governing the representation of category hierarchy in the ITC as well. The current data indicate that subordinate-level facial information is sparsely scattered within the ITC, spanning out of the ordinate-level face-selective domain rather than discretely clustering within it, as illustrated in a partially speculative schema in Figure 7*B*.

### Effects of Temporal Coherence on Representation by Spatially Summated Signals

A characteristic category-specific reduction of decoding accuracy by spatial summation was found in the early evoked time window; view and species category information were decoded with equivalent accuracy with early theta LFP in monkey H, but only the performance of species decoding was reduced with early theta ECoG (Fig. 6*A*). The results are consistent with the finding that in the early evoked period, the neural population representing species subcategories exhibits relatively smaller but more homogeneous organization than the population representing view subcategories (Fig. 5*B* top left, Fig. 5*E* left). In addition to the spatial configuration of neural activity, a temporal effect may also have contributed to the robustness of view decoding in ECoG. An analysis of temporal phase information revealed that the theta signal for the right-view face arrived at the recorded region in a less correlated manner than the center- and left-view faces (Fig. 6*C* bottom). This may have provided right-view–specific signal reduction and robust distinction across views in the spatially summated ECoG signal. We speculate that the nonlinearity mentioned in the preceding section have arisen, at least in part, from the temporal structure of IT neural responses. This interpretation is consistent with the idea that the spatial reach of the recorded neural signal depends not only on the spatial configuration but also on the temporal coherence of source signals, since phase matching of synaptic activity affects the spatial summation of the signal (Linden et al. 2011; Einevoll et al. 2013).

### Contribution of Higher Order Correlation

**Figure 8.**
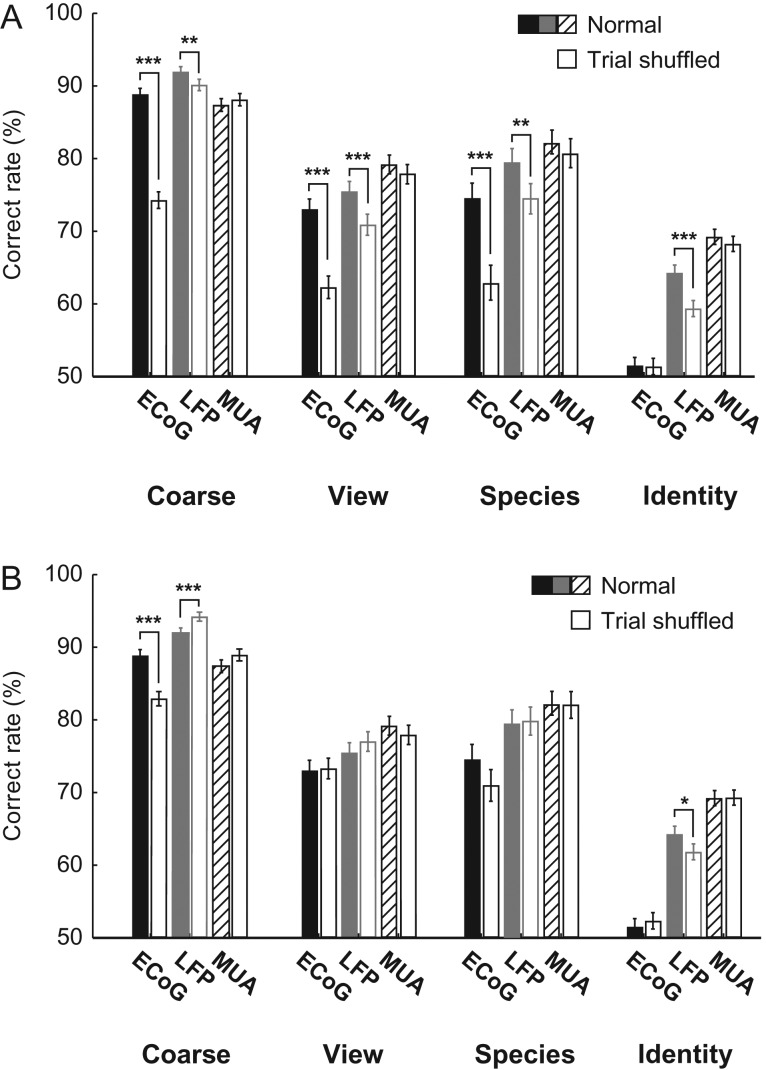
Category decoding with trial shuffling. (*A*) Decoding performance with shuffled training data and original test data. The shuffled performance shows the amount of loss when the classifier is built, neglecting a trial covariance structure in the data. (*B*) Decoding performance with shuffled training and test data. The shuffled data set resembles a case where a serially acquired data is later pooled (e.g., pooled single-unit data) for use in multivariate analysis. *P < 0.05; **P < 0.01; ***P < 0.001, Chi-squared test with Bonferroni correction for multiple comparisons.

In multichannel neural data, important information can be embedded in higher order correlation across channels (Maynard et al. 1999). To address this issue, we conducted 2 types of decoding analyses by manipulating the covariance structure of the data. In the first analysis, we trained the category classifiers with trial-shuffled data and classified the original data (Fig. 8*A*). This procedure maintains the trial average but destroys the trial-wise covariance structure of the training data. Thus, the outcome performance may reveal the amount of loss that would occur if the trial covariance was negligible in training the category classifiers. Classification performance significantly decreased compared with the original data, indicating substantial trial covariance in the ECoG/LFP data (Fig. 8*A*). Several factors may explain this covariance: 1) noise unrelated to neural activity, 2) visual stimuli–unrelated neural activity fluctuation, and 3) visual stimuli–related neural activity fluctuation. The latter 2 factors could arise from subthreshold membrane voltage fluctuations because MUA performance was not affected by the shuffling procedure. In the second analysis, we used trial-shuffled data for both training and testing of the category classifiers (Fig. 8*B*). This second data set resembles data obtained with single-unit recording experiments, where serially acquired data are pooled for use in multivariate analysis. These data may be plotted as mean response vectors but should not be plotted as trial-wise data unless zero covariance is assumed (Hung et al. 2005). The classification performance of coarse category and identity decoding in the shuffled LFP data differed significantly from the original data, and coarse category decoding in the shuffled ECoG data also differed significantly from the original data (Fig. 8*B*). These results suggest that the classification performance of simultaneously acquired LFP data might be underestimated unless taking significant information embedded in the higher order correlation across channels into account.

We observed several phenomena that cannot be explained by higher order correlation nor by temporal coherence. For example, late high-gamma LFP-based decoding performance was higher for species than view (Fig. 4*B* middle left), even though the channel-wise d′ appeared to be higher for view compared with species category (Fig. 5*B* right, 5*E* right). In addition, there was no clear difference between view and species in higher order correlations. A possible explanation is that there was more redundant information across remote LFP recording sites for view than for species category, giving rise to increased species decoding performance in multivariate decoding analysis.

### Implications for Brain–Machine Interfaces

ECoG is becoming an increasingly popular tool for brain–machine interfaces because it is associated with minimal tissue damage, long-term stability, large area coverage, and fewer ethical barriers for human applications (Schalk and Leuthardt 2011). However, its brain-decoding capability compared with that by neuronal spiking activity has not been studied in detail. The current study demonstrates that the reliability of category decoding by different recording methods depends on the type of target category. ECoG-based decoding was surprisingly reliable for coarse category information. LFPs can reliably predict multiple level categories including identity of the individual faces. This is valuable because the current method of identity decoding is not a simple discrimination of one particular stimulus image from another (Hung et al. 2005) but accounts for the generalization of personal identity regardless of viewing angle. High-classification performance of LFP-based decoding is presumable because it can detect both high-frequency local oscillations and across-area slow voltage synchronization. Although acquisition of LFP signals relies on invasive microelectrode penetration, it can be acquired stably for a long period. Overall, the current results suggest that LFP-based decoding could provide a powerful neurophysiological and prosthetic tool for reading out a wide range of targeted information from a small cortical window.

## Supplementary Material

Supplementary Data

## References

[bhx342C1] Arieli A, Grinvald A, Slovin H 2002 Dural substitute for long-term imaging of cortical activity in behaving monkeys and its clinical implications. J Neurosci Methods. 114:119–133. 11856563 10.1016/s0165-0270(01)00507-6

[bhx342C2] Belitski A, Gretton A, Magri C, Murayama Y, Montemurro MA, Logothetis NK, Panzeri S 2008 Low-frequency local field potentials and spikes in primary visual cortex convey independent visual information. J Neurosci. 28:5696–5709. 18509031 10.1523/JNEUROSCI.0009-08.2008 PMC6670798

[bhx342C3] Bell AH, Malecek NJ, Morin EL, Hadj-Bouziane F, Tootell RB, Ungerleider LG 2011 Relationship between functional magnetic resonance imaging-identified regions and neuronal category selectivity. J Neurosci. 31:12229–12240. 21865466 10.1523/JNEUROSCI.5865-10.2011 PMC3165163

[bhx342C4] Brincat SL, Connor CE 2006 Dynamic shape synthesis in posterior inferotemporal cortex. Neuron. 49:17–24. 16387636 10.1016/j.neuron.2005.11.026

[bhx342C5] Buzsaki G, Anastassiou CA, Koch C 2012 The origin of extracellular fields and currents–EEG, ECoG, LFP and spikes. Nat Rev Neurosci. 13:407–420. 22595786 10.1038/nrn3241 PMC4907333

[bhx342C6] Chang CC, Lin CJ 2011 LIBSVM: a library for support vector machines. ACM T Intel Syst Tec. 2:27.

[bhx342C7] Contreras D, Steriade M 1995 Cellular basis of EEG slow rhythms: a study of dynamic corticothalamic relationships. J Neurosci. 15:604–622. 7823167 10.1523/JNEUROSCI.15-01-00604.1995 PMC6578315

[bhx342C8] Dotson NM, Goodell B, Salazar RF, Hoffman SJ, Gray CM 2015 Methods, caveats and the future of large-scale microelectrode recordings in the non-human primate. Front Syst Neurosci. 9:149. 26578906 10.3389/fnsys.2015.00149 PMC4630292

[bhx342C9] Einevoll GT, Kayser C, Logothetis NK, Panzeri S 2013 Modelling and analysis of local field potentials for studying the function of cortical circuits. Nat Rev Neurosci. 14:770–785. 24135696 10.1038/nrn3599

[bhx342C10] Epstein R, Kanwisher N 1998 A cortical representation of the local visual environment. Nature. 392:598–601. 9560155 10.1038/33402

[bhx342C11] Haxby JV, Gobbini MI, Furey ML, Ishai A, Schouten JL, Pietrini P 2001 Distributed and overlapping representations of faces and objects in ventral temporal cortex. Science. 293:2425–2430. 11577229 10.1126/science.1063736

[bhx342C12] Helmchen F, Svoboda K, Denk W, Tank DW 1999 In vivo dendritic calcium dynamics in deep-layer cortical pyramidal neurons. Nat Neurosci. 2:989–996. 10526338 10.1038/14788

[bhx342C13] Hung CP, Kreiman G, Poggio T, DiCarlo JJ 2005 Fast readout of object identity from macaque inferior temporal cortex. Science. 310:863–866. 16272124 10.1126/science.1117593

[bhx342C14] Huth AG, Nishimoto S, Vu AT, Gallant JL 2012 A continuous semantic space describes the representation of thousands of object and action categories across the human brain. Neuron. 76:1210–1224. 23259955 10.1016/j.neuron.2012.10.014 PMC3556488

[bhx342C15] Kamitani Y, Tong F 2005 Decoding the visual and subjective contents of the human brain. Nat Neurosci. 8:679–685. 15852014 10.1038/nn1444 PMC1808230

[bhx342C16] Kanwisher N, McDermott J, Chun MM 1997 The fusiform face area: a module in human extrastriate cortex specialized for face perception. J Neurosci. 17:4302–4311. 9151747 10.1523/JNEUROSCI.17-11-04302.1997 PMC6573547

[bhx342C17] Kiani R, Esteky H, Mirpour K, Tanaka K 2007 Object category structure in response patterns of neuronal population in monkey inferior temporal cortex. J Neurophysiol. 97:4296–4309. 17428910 10.1152/jn.00024.2007

[bhx342C18] Kreiman G, Hung CP, Kraskov A, Quiroga RQ, Poggio T, Dicarlo JJ 2006 Object selectivity of local field potentials and spikes in the macaque inferior temporal cortex. Neuron. 49:433–445. 16446146 10.1016/j.neuron.2005.12.019

[bhx342C19] Kreiman G, Koch C, Fried I 2000 Category-specific visual responses of single neurons in the human medial temporal lobe. Nat Neurosci. 3:946–953. 10966627 10.1038/78868

[bhx342C20] Kriegeskorte N, Mur M, Ruff DA, Kiani R, Bodurka J, Esteky H, Tanaka K, Bandettini PA 2008 Matching categorical object representations in inferior temporal cortex of man and monkey. Neuron. 60:1126–1141. 19109916 10.1016/j.neuron.2008.10.043 PMC3143574

[bhx342C21] Ku SP, Tolias AS, Logothetis NK, Goense J 2011 fMRI of the face-processing network in the ventral temporal lobe of awake and anesthetized macaques. Neuron. 70:352–362. 21521619 10.1016/j.neuron.2011.02.048

[bhx342C22] Linden H, Tetzlaff T, Potjans TC, Pettersen KH, Grun S, Diesmann M, Einevoll GT 2011 Modeling the spatial reach of the LFP. Neuron. 72:859–872. 22153380 10.1016/j.neuron.2011.11.006

[bhx342C23] Majima K, Matsuo T, Kawasaki K, Kawai K, Saito N, Hasegawa I, Kamitani Y 2014 Decoding visual object categories from temporal correlations of ECoG signals. Neuroimage. 90:74–83. 24361734 10.1016/j.neuroimage.2013.12.020

[bhx342C24] Matsuo T, Kawasaki K, Osada T, Sawahata H, Suzuki T, Shibata M, Miyakawa N, Nakahara K, Iijima A, Sato N, et al 2011 Intrasulcal electrocorticography in macaque monkeys with minimally invasive neurosurgical protocols. Front Syst Neurosci. 5:34. 21647392 10.3389/fnsys.2011.00034 PMC3103840

[bhx342C25] Maynard EM, Hatsopoulos NG, Ojakangas CL, Acuna BD, Sanes JN, Normann RA, Donoghue JP 1999 Neuronal interactions improve cortical population coding of movement direction. J Neurosci. 19:8083–8093. 10479708 10.1523/JNEUROSCI.19-18-08083.1999 PMC6782478

[bhx342C26] Mitzdorf U 1985 Current source-density method and application in cat cerebral cortex: investigation of evoked potentials and EEG phenomena. Physiol Rev. 65:37–100. 3880898 10.1152/physrev.1985.65.1.37

[bhx342C27] Mitzdorf U 1987 Properties of the evoked potential generators: current source-density analysis of visually evoked potentials in the cat cortex. Int J Neurosci. 33:33–59. 3610492 10.3109/00207458708985928

[bhx342C28] Nakahara K, Adachi K, Kawasaki K, Matsuo T, Sawahata H, Majima K, Takeda M, Sugiyama S, Nakata R, Iijima A, et al 2016 Associative-memory representations emerge as shared spatial patterns of theta activity spanning the primate temporal cortex. Nat Commun. 7:11827. 27282247 10.1038/ncomms11827 PMC4906394

[bhx342C29] Quiroga RQ, Reddy L, Kreiman G, Koch C, Fried I 2005 Invariant visual representation by single neurons in the human brain. Nature. 435:1102–1107. 15973409 10.1038/nature03687

[bhx342C30] Rosch E 1978 Principles of categorization In: Rosen E, Lloyd BB, editors Semantic factors in cognition. Hillsdale, NJ: Erlbaum p. 137–168.

[bhx342C31] Sato T, Uchida G, Lescroart MD, Kitazono J, Okada M, Tanifuji M 2013 Object representation in inferior temporal cortex is organized hierarchically in a mosaic-like structure. J Neurosci. 33:16642–16656. 24133267 10.1523/JNEUROSCI.5557-12.2013 PMC6618530

[bhx342C32] Schalk G, Leuthardt EC 2011 Brain-computer interfaces using electrocorticographic signals. IEEE Rev Biomed Eng. 4:140–154. 22273796 10.1109/RBME.2011.2172408

[bhx342C33] Shimazaki H, Shinomoto S 2010 Kernel bandwidth optimization in spike rate estimation. J Comput Neurosci. 29:171–182. 19655238 10.1007/s10827-009-0180-4 PMC2940025

[bhx342C34] Sugase Y, Yamane S, Ueno S, Kawano K 1999 Global and fine information coded by single neurons in the temporal visual cortex. Nature. 400:869–873. 10476965 10.1038/23703

[bhx342C35] Takeuchi S, Ziegler D, Yoshida Y, Mabuchi K, Suzuki T 2005 Parylene flexible neural probes integrated with microfluidic channels. Lab Chip. 5:519–523. 15856088 10.1039/b417497f

[bhx342C36] Tamura H, Tanaka K 2001 Visual response properties of cells in the ventral and dorsal parts of the macaque inferotemporal cortex. Cereb Cortex. 11:384–399. 11313291 10.1093/cercor/11.5.384

[bhx342C37] Toda H, Suzuki T, Sawahata H, Majima K, Kamitani Y, Hasegawa I 2011 Simultaneous recording of ECoG and intracortical neuronal activity using a flexible multichannel electrode-mesh in visual cortex. Neuroimage. 54:203–212. 20696254 10.1016/j.neuroimage.2010.08.003

[bhx342C38] Tsao DY, Freiwald WA, Knutsen TA, Mandeville JB, Tootell RB 2003 Faces and objects in macaque cerebral cortex. Nat Neurosci. 6:989–995. 12925854 10.1038/nn1111 PMC8117179

[bhx342C39] Tsao DY, Freiwald WA, Tootell RBH, Livingstone MS 2006 A cortical region consisting entirely of face-selective cells. Science. 311:670–674. 16456083 10.1126/science.1119983 PMC2678572

[bhx342C40] Tsao DY, Moeller S, Freiwald WA 2008 Comparing face patch systems in macaques and humans. Proc Natl Acad Sci USA. 105:19514–19519. 19033466 10.1073/pnas.0809662105 PMC2614792

[bhx342C41] Tsunoda K, Yamane Y, Nishizaki M, Tanifuji M 2001 Complex objects are represented in macaque inferotemporal cortex by the combination of feature columns. Nat Neurosci. 4:832–838. 11477430 10.1038/90547

[bhx342C42] Vapnik V 1998 The Support Vector method of function estimation. Nonlinear Modeling. 55–85.

[bhx342C43] Vindiola M, Wolmetz M 2011 Mental encoding and neural decoding of abstract cognitive categories: a commentary and simulation. Neuroimage. 54:2822–2827. 20974265 10.1016/j.neuroimage.2010.09.091

[bhx342C44] Wang G, Tanaka K, Tanifuji M 1996 Optical imaging of functional organization in the monkey inferotemporal cortex. Science. 272:1665–1668. 8658144 10.1126/science.272.5268.1665

