# Supplementary material for: Heterogeneous Redistribution of Facial Subcategory Information Within and Outside the Face-Selective Domain in Primate Inferior Temporal Cortex
Source: Cereb Cortex. 2018 Jan 10;28(4):1416–31. doi: 10.1093/cercor/bhx342 (PMC6093347; doi:10.1093/cercor/bhx342)
Supplement: Supplementary Data [file bhx342supplementarymaterial_cc_v3.docx]

# Supplementary Material


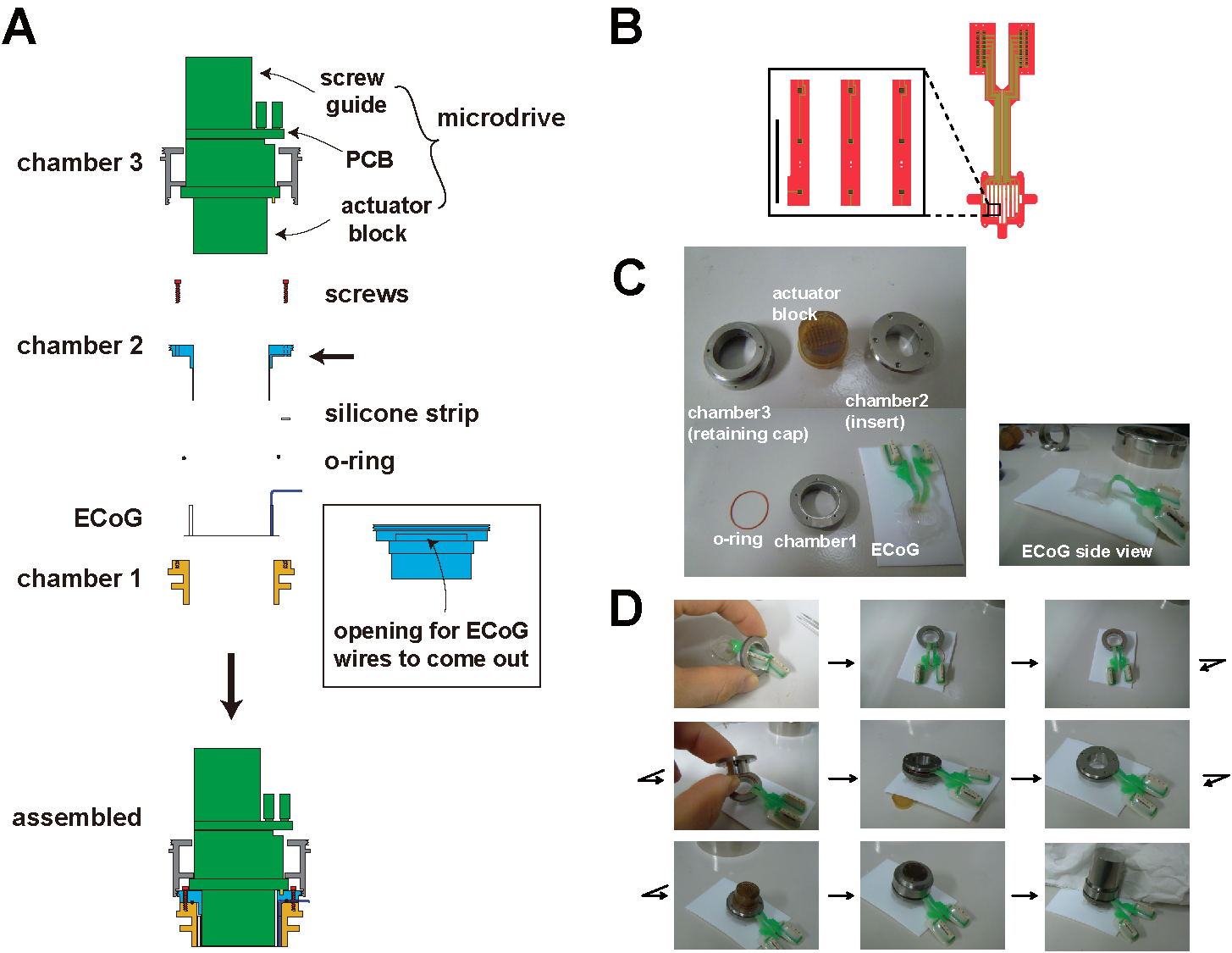


**Figure S1**

**Device assembly procedure.**

(A) Recording device in projected and sectioned views. Parts excluding the microdrive (green) are displayed in cross-section. (Top) is the partially assembled state, in which the microdrive for microelectrodes is fully assembled; however, the two bottom chambers, the ECoG electrode, and a few other small parts are shown separately. The screws (red) firmly attach chamber 2 onto chamber 1, with an o-ring providing a water-tight seal. (Inset) shows the projected side view of chamber 2, showing the position of the concave path used to pass the ECoG wires. A silicone strip is placed on top of the ECoG wires to secure the seal. (Bottom) shows the assembly of all the parts in the left figure. The names of the major parts correspond to those indicated in Figure 2. (B) 2D schematic drawing of the original ECoG. ECoG is prepared using MEMS technology (see Materials and methods for details). The yellow lines represent gold wires. The brown squares (blown-up image) are ECoG contacts, and the region in red depicts the Parylene-C film, which is actually transparent (see A). Note that the ECoG gold wires are covered with Parylene-C film for electrical insulation and that only the electrode contacts are exposed. (C) ECoG and microelectrode array assembled in dry condition according to the steps used in actual chronic assembly. ECoG is placed on the cortical surface, with the side brim of the artificial dura inserted beneath the cut end of the dura during surgical placement. The titanium chamber and o-ring are placed by passing ECoG connectors through the center holes. Chamber 2 is inserted into the upright tubal part of ECoG and attached to chamber 1. The microdrive is inserted into chamber 2, and chamber 3 firmly screws onto chamber 2. The microdrive, screw guide, and chamber 3 are shown separately for clarity but are preassembled before mounting during the actual implant process (A). See Methods for further detail. (D) Electrode array parts. ECoG is shielded with silicone rubber (green) for protection of the lead wire section located between the electrode contact and the connectors. The white plastic sheet beneath ECoG is not part of the recording system. Scale bar, 1 mm (B, blown up).

**
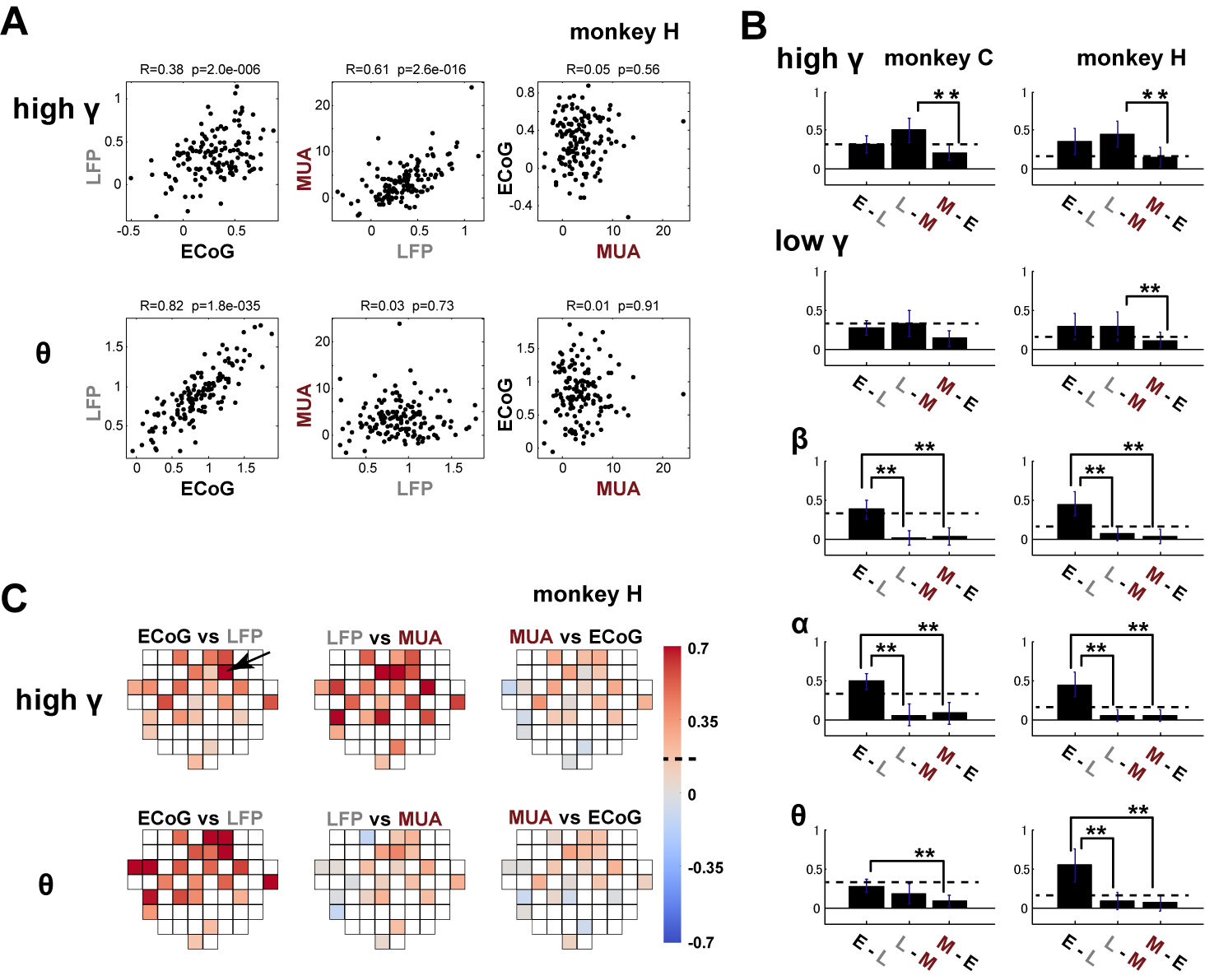
Figure S2**

**Correlation of stimulus selectivity across measurements at each recording site.**

Stimulus selectivity relationships between recording methods. (B) Population stimulus selectivity relationships between recording methods averaged across recording sites. “E–L” is the mean correlation value of ECoG–LFP, shown with the SD bar; “L–M” and “M–E” are those of LFP–MUA and MUA–ECoG, respectively. Dotted lines indicate statistical significance (*p*< 0.05) with the given number of stimuli (35 stimuli for monkey C and 144 stimuli for monkey H) of the pooled correlation value. Black asterisks indicate significant differences between correlation values for each comparison. **p* < 0.05; ***p* < 0.01, Kolmogorov–Smirnov test corrected for multiple comparisons. (C) Color-coded correlation values between each recording method plotted at each site for monkey H. The dashed line on the color bar indicates the statistical significance (*p*< 0.05) of the pooled correlation value.

**
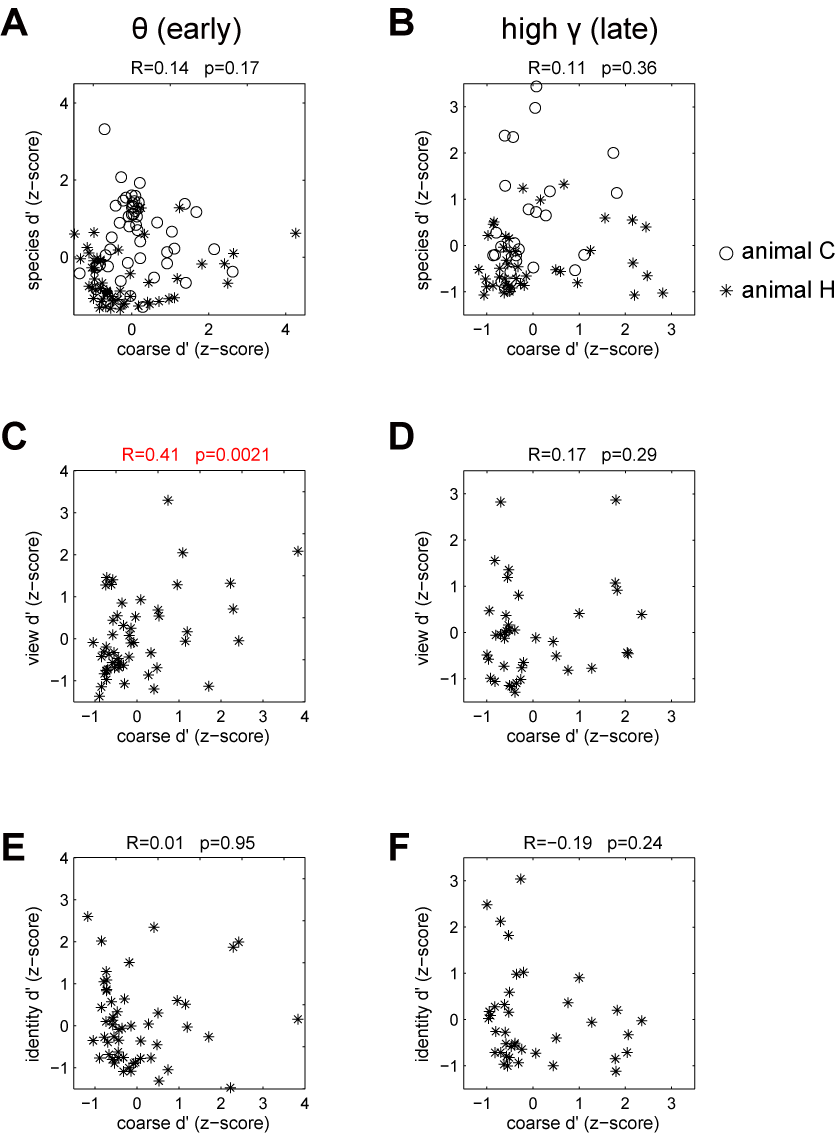
**

**Figure S3**

**Correlation of d' value across distinct types of categorization at each recording site.**

d' relationships of coarse versus species categorization (A), coarse versus view categorization (B), and coarse versus identity categorization (C).

**
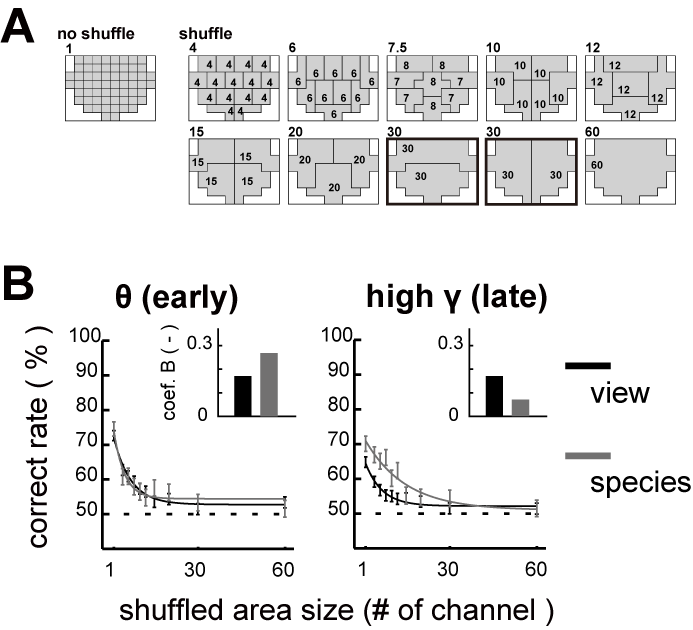
**

**Figure S4**

**Effect of spatial shuffling on view and species category decodings in monkey H.**

(A) Schematic maps showing different levels of spatial shuffling. The gray subsections indicate the areas in which spatial shuffling of the data was performed. The numeral in each subarea indicates the number of channels shuffled within the subarea. The numeral in bold above each map indicates the average number of shuffled channels in the subareas. Note that the decoding performance for 30-channel shuffling is obtained by averaging the data from the two different shuffle maps indicated by bold frames in bottom row. (B) View (black) and species (gray) decoding performance (y) using early theta (left) and late high-gamma (right) LFP responses with spatial shuffling, plotted against the size of the subarea used for shuffling (x). Data were fit with a curve y = A exp(−Bx) + C, where A, B, and C are positive constants. (Insets) The decay constant “B” reflecting the speed of decline for the fit curve, i.e., the sensitivity of the category information against spatial shuffling. Error bars indicate the 95% confidence limit.
